# Neonatal CD19^+^B220^lo^ cells sense microbiota via TLR2/4 activation driving proliferation and differentiation

**DOI:** 10.3389/fimmu.2026.1838044

**Published:** 2026-06-19

**Authors:** Carolina Ruiz-Sánchez, Isabel Cortegano, Mercedes Rodríguez, Rodrigo Sanchez-Tarjuelo, Alejandro Arrabal, M. Carmen Prado, Mario Alia, M.Pilar Jiménez, Victor Manuel Lopez Molina, Sara Monzón, Victoria López-Alonso, Belén de Andrés, Maria-Luisa Gaspar

**Affiliations:** 1 Immunobiology Unit, Immunology Department, Centro Nacional de Microbiología (CNM), Instituto de Salud Carlos III (ISCIII), Majadahonda, Spain; 2 Flow Cytometry Unit, Central Core Laboratories, ISCIII, Majadahonda, Spain; 3 Genomic Unit, Central Core Laboratories, ISCIII, Majadahonda, Spain; 4 Bioinformatic Unit, Central Core Laboratories, ISCIII, Majadahonda, Spain; 5 Computational Biology Unit, Chronic Disease Research Program, ISCIII, Majadahonda, Spain

**Keywords:** B lymphocytes, IgH repertoires, cytokines, immunoglobulins, lung neonatal immune response, microbiota, B220lo cells

## Abstract

Neonatal respiratory infections remain a leading cause of mortality and morbidity worldwide, yet there is still little information about the role of B cell populations in the respiratory tract. CD19^+^B220^lo^ are B cells that are found in neonatal lungs, and antibiotic treatment of pregnant females and their offspring reduced the number of these cells and modified their VH immunoglobulin repertoire. Activation *in vitro* by the TLR2 (PAM3 and FSL1) and TLR4 (LPS) ligands induced proliferation and differentiation of neonatal B cells in the lung and spleen, generating CD138^+^ cells and releasing IgM, IgG1 and small amounts of IgA, as well as several cytokines. A higher frequency of CD138^+^ cells was observed within both purified CD19^+^B220^lo^ and CD19^+^B220^hi^ populations following *in vitro* TLR2/TLR1 stimulation with PAM3. The lung cultures adopted an inflammatory cytokine profile (TNF-α, IL1-α, IL6), as opposed to the regulatory profile (IL10) of spleen cultures. Taken together, these findings support the conclusion that neonatal lung and spleen CD19^+^B220^lo^ cells can sense the microbiota via both TLR2 and TLR4, thereby promoting their activation and differentiation.

## Introduction

The lung is an organ specialized in gas exchange that is a target for many pathogens and environmental insults. Its role as a defensive barrier is highly important since the respiratory epithelium, with the mucus and the antimicrobial molecules (including peptides and surfactant proteins), acts as a physical barrier while allowing gas exchange (1, 2). During neonatal life the lung is also an organ in which early immune responses are shaped by the first microbial and environmental exposures, making the study of innate and adaptative immunity in this context essential for understanding the establishment of long-term pulmonary immune homeostasis and responsiveness. Thus, it is highly relevant to explore the role of adaptative cells such as B cell populations developing in the neonatal lung.

Cells of the innate immune system (IS) in the lung play an important role in controlling the invasion of microorganisms in this organ through highly regulated local interactions in response to lung pathogens (3–6). The cells of the innate IS use “pattern recognition receptors” (PRRs) to recognize pathogens motifs known as “pathogen-associated molecular patterns” (PAMPs) (7, 8). The activation of PRRs by PAMPs, or by endogenous signals of danger, triggers inflammatory responses that are designed to eliminate the pathogens.

Several families of PRRs exist, one of which is the Toll-like receptor (TLR) family that is composed of 13 receptors in the mouse (9), type I transmembrane proteins that are located in the plasma and endosomal membranes (10). Among the TLRs in the plasma membrane, TLR2 recognizes PAMPs of Gram^+^ bacteria (lipoproteins, lipomannan and lipoteichoic acids) and to a lesser extent, lipopolysaccharide (LPS) of Gram^-^ bacteria, while TLR4 mainly recognizes LPS. Two major signaling pathways are involved in TLR activation (10), one of which is used by all TLRs except TLR3, and it depends on MyD88 (myeloid differentiation primary response gene 88). In addition, TLR3 and TLR4-mediated IFNβ responses depend on signaling through TRIF (TIR-domain-containing adaptor protein inducing IFNβ). TLRs are expressed in a wide range of tissues and cell compartments around the body, including mucosal cells like those found in the respiratory airway and lungs (1, 2), as well as in lympho-myeloid cells (11).

Our organism is constantly exposed to the microbiota that colonizes the skin and the mucous membranes (12), the composition of which influences the course of certain metabolic diseases and infections. The local respiratory microbiota has an important influence on the correct resolution of lung infections (13). As such, the TLR4 and MyD88 activation pathways are essential to control sepsis by Gram^+^ and Gram^-^ bacteria (14–16). Furthermore, TLR signaling in the lung is involved in the activation of epithelial cells, alveolar macrophages, myeloid-derived suppressor cells, and neutrophils, cells that play a fundamental role in eliminating pathogens (17–23). The adaptive IS (T and B cells) mediates the lung responses designed to eliminate pathogens also through PRR signaling, in addition to their specific activation through their antigen specific receptors (TCR and BCR) (24–27). In recent years, lung B cell responses have been approached in respiratory infection models, such as influenza (28) and distinct subsets of lung-resident B cells have been identified (29). Significantly, the effectiveness of the lung defenses varies with age (30), shifting from defective responses in the newborn (31) to immune senescence that drives an “inflammageing” state in aged individuals (32).

It was postulated that the IS is “multilayered”, with innate lymphoid cells (ILCs) connecting innate cells with adaptive ones (33, 34). ILCs include both B lymphocytes (B1 and marginal zone B cells -MZB) and T lymphocytes (Tγ_δ_ lymphocytes, natural killer T cells), which share characteristics of the innate and adaptive IS, and exert regulatory properties (35, 36). These cells have little diversity in their BCRs or TCRs, and they are characterized by a preferential localization in particular niches: celomic cavities for B1 lymphocytes, marginal zone of the spleen for MZB lymphocytes, and mucosal associated tissues for Tγ_δ_ lymphocytes. In this way, the ILC layer would provide specific, albeit low affinity, elements that are prepared to respond in a rapid and limited fashion (34, 37, 38). Together with their topographic location, this converts these cells into the first line adaptive response to pathogens (39).

Murine B1 lymphocytes differ in several ways from conventional B2 CD19^+^CD45R/B220^hi^ B lymphocytes (from now on B220^hi^), as they display a specific phenotype based on their membrane antigens (CD19^+^IgM^++^CD45R/B220^lo^CD23^-^CD43^+^CD11b^+^CD5^+/-^), they are mostly located in the peritoneum and the pleural cavity, and they secrete multi-specific and low affinity natural IgM antibodies with relatively conserved variable regions (40). These cells are absent in Btk^-/-^ mice, and they respond preferentially to T-independent type II antigens. Two subsets of B1 cells have been identified based on their CD5 expression, B1a (CD5^+^) and B1b (CD5^-^), which are generated in distinct waves and have differential signaling thresholds (41). B1 cells secrete cytokines like IL10 spontaneously (42) and in models of sepsis, B1 cells produce innate response activator (IRA) B cells that secrete GM-CSF (43). In the mouse, B1 lymphocytes originate in the dorsal aorta/para-aortic splanchnopleura and in the liver of the embryo (33, 44). A CD19^+^B220^lo^CD5^+/-^ B cell progenitor population was also identified in the liver of the mouse embryo (45). In the adult, B1 cells are maintained by self-renewal (46), although B1 progenitors (B220^lo^IgM^-^VpreB^+^λ5^+^RAG1^+^) in mice have been identified in the adult peritoneal cavity, of which B1 cells were depleted by daily peritoneopheresis (47). Also, CD11b^-^ B1 cells in peritoneum have been identified as progenitors of CD11b^+^ B1a cells, which generate all subsets of peritoneum B1 cells upon cell transfer (48), and that could be referred as B1c cells (49), although it is most possible that they constitute a developmental and/or functional subdivision of B1 cells (48). After migration to the spleen, CD11b may be downregulated, and indeed, it was described that splenic B1a cells were CD11b^-^ (50, 51). Furthermore, a subset of CD19^+^B220^-^IgM^+^ light chain (IgL) negative cells that may represent B1 cell progenitors were found in the adult spleen (52, 53). Moreover, in the adult spleen, CD19^+^B220^lo^IgL^+^CD11b^-^CD5^+/-^ (from now on B220^lo^) cells were detected, which were Btk-dependent and of embryonic origin. These cells that are closely linked to splenic B1 cells (50, 51), represent a heterogeneous population of IgM^+/-^IgL^+^ cells that produce IgG, IgA and IL10 under basal conditions and when activated by LPS (54, 55).

B lymphocytes express TLRs, through which they can be effectively activated and differentiated (56, 57). Here, the role of TLR2 and TLR4 in newborn B220^lo^ cells and B220^hi^ cells was assessed in the lung and spleen of WT, MyD88^-/-^, TLR4^-/-^ and TLR2^-/-^TLR4^-/-^ mice. Higher frequencies of B220^lo^ cells were found in the lung than the spleen of WT mice from early life, these cells representing a heterogeneous population containing immature and differentiated B cells and share a B1-like phenotype. The contribution of microbiota to perinatal B cell development was assessed by evaluating the effects of antibiotic treatment, which produced a significant decrease in B220^lo^ cells and a change in their VH repertoires. Furthermore, fewer B220^lo^ cells were registered in the absence of MyD88, TLR4 and both TLR2 and TLR4. The TLR2 and TLR4 ligands induced MyD88-dependent proliferation *in vitro*, the differentiation of perinatal lung and spleen cells, and the secretion of immunoglobulins and cytokines. Moreover, a higher IL6/IL10 pro-inflammatory ratio was obtained from lung cell cultures than from spleen cells.

## Materials and methods

### Experimental models

C57BL/6J mice (BL6) were purchased from Charles River and B6.129P2(SJL)-Myd88tm1.1Defr/J (MyD88^-/-^), C57BL/10J (BL10) and TLR4-B10.129-Tlr2tm1Kir/J (TLR4^-/-^) mice were kindly provided by Dr Julian Pardo (Universidad de Zaragoza). The double KO mice were provided by Dr. Manuel Fresno (Centro de Biología Molecular Severo Ochoa). The animals were kept under pathogen-free conditions at the Carlos III Health Institute (Madrid) animal facility (Nb ES280800000015). Animals were sacrificed with carbon dioxide or by decapitation in the case of neonates. All procedures were performed in agreement with approved protocols and guidelines established by the Ethics and Animal Welfare Committee of the Carlos III Health Institute, and with authorization of the Community of Madrid (PROEX numbers 110/15, 021/18, 282.4/20).

### Administration of antibiotics and genomic PCR for microbiota analysis

Ampicillin (1 g/L), Neomycin (0.5 g/L) and Erythromycin (10 mg/L, all from Sigma-Aldrich Merck) were administered to pregnant females *ad libitum* in the drinking water from gestational day 11.5 until the end of the experiment (58, 59). At E18 the females were separated into individual cages. After birth and until sacrifice, the neonates were administered a daily solution of 20 µL antibiotics (2 µg/µL Ampicillin and 1 µg/µL Neomycin in water) using a micropipette to deliver the mix orally. Untreated pregnant females and neonates (the latter administered with 20 µL of water) were included in parallel as controls in each experiment. Once sacrificed, the lungs and spleens were dissected, and the cell suspensions were prepared as indicated above. Supernatants from the cell pellets of lungs of antibiotic treated and control D7 pups were recovered and frozen at -20°C. Feces from the cages of antibiotic treated and control mice were collected the day the neonates were sacrificed. DNA was obtained from the frozen supernatants and the feces using the QiAmp Fast DNA Stool Mini kit (Qiagen). PCR of the variable V3 and V4 regions of the rDNA 16s was performed by Dreamgenics S.L. (Oviedo, Spain), and the sequencing was performed by Macrogen (Seoul).

### Sample preparations

The lung and spleen were dissected out from adult or neonatal mice and cut into small pieces in Dulbecco’s phosphate-buffered saline (PBS: BioWhittaker, Lonza Group Basel, Switzerland). Cell suspensions were prepared by mechanical dissociation of individual or pooled neonatal organs of the same age, or from adult mice, and the cells were filtered through a 40 µm pore cell strainer (BD Biosciences) in PBS containing 2.5% fetal calf serum (FCS/PBS, Gibco Thermo Fisher Scientific, Waltham MA USA) before they were counted in a Neubauer chamber. A schematic representation of the analyses performed thereafter is displayed in **Supplementary Figure 1**.

### Flow cytometry and cell sorting

Cell suspensions were incubated for 10 min at 4 °C in blocking solution (Fc block: BD Biosciences Inc., San Jose, CA, USA) and they were then stained by incubating for 20 min at 4 °C with monoclonal antibodies (mAbs). All the mAbs and isotype controls used are indicated in **Supplementary Table 1**. For staining of *ex vivo* obtained cell suspensions mAbs against CD19, B220, CD11b, CD5, IgM, IgD, MHCII, CD138, CD93, CD43, CD44 and CD9 diluted in 2% FCS/PBS, before adding 10 mM propidium iodide (PI) to discard dead cells and cell debris, combined with forward scatter (FSC) gating versus side scatter (SSC). For staining of cell suspension from cultured cells mAbs against CD19, B220, and CD138 were used. To assess IL6 and IL10, cultured cells were incubated with the Live/Dead Fixable Aqua Dead Cell Stain Kit (Invitrogen) in PBS for 20 min at 4 °C, and stained with mAbs against CD19, B220, CD138, CD3 and CD11b. These cells were then fixed and permeabilized using the Cytofix/Cytoperm kit (BD Biosciences), prior to intracellular staining with PE labelled anti–IL-6, anti-IL10 or an isotype control, as described previously (55). Cell suspensions were analyzed on a FACS Fortessa X20™ cytometer (BD Biosciences), using the FlowJo v10.7 software package (Tree Star Inc., Ashland, OR, USA) and FCS Express De NOVO software for data analysis. Cell suspensions of FACS-purified (over 95% purity) B220^lo^ and B220^hi^ cells from lung and spleen were prepared. Briefly, a Ficoll density gradient of lung cell suspensions was performed to obtain cells in the lymphoid cell interphase (Cedarlane Labs, Ontario). The lymphoid lung cell suspensions and the spleen cell suspensions (a pool of three per sample) were then blocked and stained with mAbs against CD19-PE and B220-PE-Cy7, to purify B220^lo^ and B220^hi^ cells on a FACSAria-II (BD Biosciences) cell sorter.

### Analysis of the VH immunoglobulin repertoire

The IgH repertoire analysis of sorted B220^lo^ and B220^hi^ cells from neonatal antibiotic treated and control spleen samples was performed as described previously (60). Briefly, RNA was extracted using the NucleoZOL Reagent, as indicated by the manufacturer, and cDNAs were synthesized using the oligo-(dT) primer and avian myeloblastosis virus reverse transcriptase (Promega, Madison, WI, USA). PCR amplifications of the VDJCμ rearranged transcripts were performed with 1U Supreme NZYTaq II DNA polymerase (NZYTech, Portugal) in a PTC-200 DNA Engine cycler (Bio-Rad), using a degenerate VH primer (covering the IGHV1 to IGHV7 families; 5’-AGGTSMARCTKCWSSAGTCWGG-3’) and a CHμ-specific primer, (5’-GGGAAATGGTGCTGGGCAGGAA-3’) as described previously (61). Tags were used to add a barcode and Illumina adapters, and amplicons were prepared with the Nextera XT Index kit v2 (Illumina Inc, San Diego, CA, USA), as described by the manufacturer. The products were sequenced on the MiSeq platform (Illumina) and the bioinformatics processing included preprocessing with VDJPipe (62), sequence fusion by filtering with a minimum average quality of 35 and a maximum homopolymer of 20, and collapsing into files with total Fasta sequences using the VDJ-server Release 1.1.2 (https://vdjserver.org/). Once the sequences were successfully matched, they were submitted to IMGT/HighV-Quest Release 3.4.17 (63) for annotation of the CDR3 and full-length VDJCμ regions. The IMGT output files were analyzed in ARGalaxy and IMGT-clonotype for the analysis of the mutation frequencies (64, 65). To analyze V–J gene segment pairing patterns, IGHV and IGHJ gene calls were determined. Raw clonotype count tables were imported into RStudio (IDE version 2025.09.1 + 401 (66) and analyzed with R version 4.4.1 (67). Additionally, the following R packages were employed: readxl v1.4.5 (68) for data import, dplyr v1.1.4 (69) for data wrangling, and circlize v0.4.16 (70), for the generation of chord diagrams (circos plots), which were created to visualize the connectivity between IGVH and IGJH gene families. For the creation of these plots, IGHV and IGHJ genes were grouped by their family number, and each V-J pair count was calculated, given the clonotypes reported in the counts table. A fixed color scheme was applied to all plots. All sequencing data generated in this study are available in the European Nucleotide Archive (ENA) under the project accession PRJEB109791 (study accession ERA35989486).

### Cell culture

Neonatal (D7) cell suspensions from the spleen and lung were cultured under sterile conditions at 37 °C in 5% CO_2_ with 95% humidity. Before plating, the cells were incubated with a cell tracer in PBS (2.5µM: Violet Cell trace Invitrogen) and an aliquot was analyzed by flow cytometry at t = 0 to obtain the initial signal. The cells were seeded at 1 x 10^6^/well (spleen cells) and 2 x 10^6^/well (lung cells) in 48-well plates (Thermo Fisher Scientific), in 300 µL of RPMI 1640 supplemented with 10% FCS, 2 mM L-Glutamine, 10 mM non-essential amino acids, 100 U/ml Penicillin-Streptomycin (all from Lonza BioWhittaker), and 50 µM 2-β-mercaptoethanol (Sigma-Aldrich Merck, Darmstadt, Germany). Purified B220^lo^ and B220^hi^ cells were seeded at 0.1-0.2 x 10^6^/well in 96-well plates and cultured under the indicated conditions. The TLR ligands for TLR1/TLR2 (Synthetic triacylated lipoprotein Pam3CSK4 -PAM3-, 1 μg/mL: InvivoGene, Toulouse France), TLR2/TLR6 (Synthetic diacylated lipoprotein FSL1, 1 μg/mL: InvivoGene), and TLR4 (Lipopolysaccharide LPS L3012, 25 μg/mL: Sigma-Aldrich Merck) were added to the cultures and the cells were observed daily under a Leica DMI3000B microscope. After 72 hours in culture, the cells and the supernatants were collected and stained as indicated above. To detect the intracellular IL6 and IL10, lung and spleen preparations were seeded at 4 x 10^6^ cells/well, and Brefeldin A (10 μg/mL: Sigma-Aldrich Merck) was added to these cultures during the final 3.5 h before they were collected for flow cytometry studies.

### Cytokine determination

A 13-multiplex Cytometric Bead Array (IL1α, IL1β, IL6, IL10, IL12p70, IL17A IL27, IL23, IFNβ, IFNγ, TNFα, MCP1/CCL2 and GM-CSF: Biolegend, CA, USA) was used to quantify the cytokines in the supernatants of the cell cultures. Measurements were made in duplicate according to the manufacturer´s instructions and the samples were analyzed on a Fortessa X20™ cytometer using the LEGENDplex™ Data Analysis Software Suite. The calibration curves were above r^2^ 0.97.

### Immunoglobulin determination

IgM, IgG1 and IgA were measured in the supernatants of the cell cultures by ELISA. Briefly, 96-well plates (Nunc, Rochester NJ, USA) were coated with unlabeled goat-anti mouse immunoglobulins (10 µg/mL: Southern Biotechnology Birmingham AL, USA) and blocked with 0.5% gelatin (Sigma-Aldrich Merck) in PBS. Supernatants (50 μL) were added to the wells in duplicate and incubated for 2 hr at 37 °C. After washing, the plates were incubated for 1 hr at 37 °C with biotinylated goat anti-mouse-IgM, anti-mouse-IgG1, or anti-mouse IgA (all from Southern Biotechnology) in 0.5% Gelatin 1% Tween 20 in PBS, and subsequently, for 30 min at 37 °C with streptavidin-peroxidase (Biolegend). The ELISA plates were revealed with 0.5 M O-phenylenediamine in 0.1M citrate buffer/0.2M Na_2_HPO_4_ and the reaction was stopped with 3N H_2_SO_4_ (all from Sigma-Aldrich Merck). OD values were obtained at 450 nm and standard curves were generated using purified IgM (clone MM-30: Biolegend), IgG1 (clone MB86, in house (71)) and IgA (clone M18-254: Becton BD Biosciences). The immunoglobulin concentrations were calculated using GraphPad Prism 5.0 software.

### *In vivo* immunization with S. pneumoniae

The bacterial 1195 strain (serotype 3, ST260 genotype), a clinical isolate that we have used previously (16), was grown to mid-log growth phase in Todd-Hewitt broth containing 0.5% yeast extract. Aliquots were prepared in 25% glycerol at a concentration of 10^8^ cells/ml and stored at -80 °C. The day of the experiment, an aliquot was thawed and heated at 60 °C for 1 hr to obtain the preparation of HK-SPN for intranasal administration to D7 neonates (10 μL/mice of HK-SPN solution containing 2x10^5^ SPN/µL in saline buffer). Complete litters were used for immunization or as controls (receiving saline solution), and they were sacrificed 3 days later.

### Statistical analysis

Statistical analysis of the results was performed using GraphPad Prism software (GraphPad Software, v9) and the data are presented as the mean ± standard error of the mean (SEM). For comparative analyses, tests of normality were performed (D’Agostino-Pearson, Shapiro-Wilks, and Kolmogorov-Smirnov normality tests) and the One-way ANOVA with *post-hoc* Tukey and parametric unpaired or paired Student’s t test were applied. Statistical significance is considered when *P < 0.05, **P < 0.01, ***P < 0.001 or ****P < 0.0001.

## Results

### B220^lo^ B lymphocytes are present in the lung

**Figure 1:**
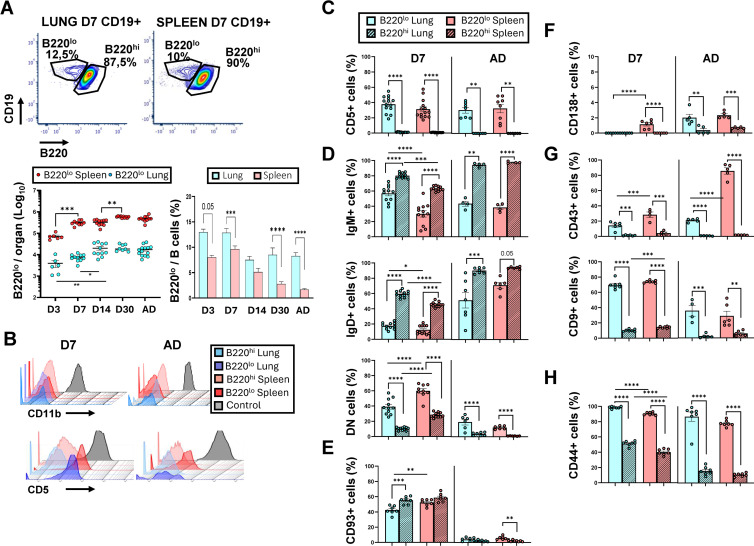
CD19^+^B220^lo^ B lymphocytes accumulate in the lung. Cell suspensions of lung and spleens were stained with mAbs CD19-BUV395, B220-APC, CD5-AF647, CD11b-PE-Cy7, IgM-FITC and IgD-BV605, or with CD19-BV421, B220-PE-Cy7, MHC-II-FITC, CD93-APC, CD138-BV711, CD44-BV510 and CD43-PE or CD9-PE. Cells were thereafter analyzed by flow cytometry on electronically gated B220^lo^ and B220^hi^ cells, excluding dead cells with the live-dead reagent BV510 or with PI, **(A)** Upper panel, representative flow cytometry contour plots of CD19 vs B220 on gated CD19^+^ B lymphocytes obtained from lung and spleen samples at D7 after birth. The numbers in the plots are the frequencies of the B220^lo^ and B220^hi^ CD19^+^ populations. Middle left, the absolute number of B220^lo^ cells in the lung and spleen from D3 to adult (AD) samples represented as dots (blue, lung; red, spleen), and the mean ± SEM (n = 6-16) as horizontal and vertical lines, respectively. Middle right, the bars represent the frequency as the mean ± SEM (n = 3-16) of B220^lo^ over B cells from D3 to AD mice in the lung (blue) and spleen (red) samples. **(B)** Shown are representative overlay histograms of the staining with CD11b and CD5 in electronically gated B220^lo^ and B220^hi^ cells from lung and spleen samples. **(C–H)** The bar plots show the percentage of CD5+, IgM+, IgD+, IgM-IgD-, CD93+, CD138+, CD43+, CD9+ and CD44+ cells on electronically gated B220^lo^ and B220^hi^ cells as in **(B)** The bars represent the mean ± SEM and the individual sample values displayed as dots. **(C, D)** n = 14 for D7 and 6–8 for AD samples. **(E–H)** n = 6–7 for D7 samples and 4–7 for AD samples. The data were compared with One-way ANOVA with *post-hoc* Tukey correction: *P < 0.05; **P < 0.01; *** *P* < 0.005; ****P < 0.0001.

We previously found that B220^lo^ B cells are mainly produced during embryonic-fetal life and that they are found in the spleen from day 7 post-partum (D7) to adulthood (54, 55). We extended here this data to the lung after birth. B220^lo^ cells were found from birth until adulthood in both the spleen and lung (**Figure 1A**), albeit in the latter organ, representing the total number of these cells one log less than in the spleen (**Figure 1A** bottom). However, higher numbers of B220^lo^ cells relative to total B cells were found in lungs than in spleens (**Figure 1A**, bottom right), and the ratio of B220^lo^/B cells declined in both organs from D7 onwards, albeit more strongly in spleen and hence with greater differences between lungs and spleens when analyzed in adulthood.

A more detailed phenotypic characterization of B220^lo^ B cells in lung and spleen at D7 and adult samples was performed (**Figure 1**; **Supplementary Figure 2**). These analyses confirmed our previous results on splenic adult B220^lo^ cells showing that both populations were consistently CD11b negative (at D7 and adult), distinguishing them from canonical peritoneal B1a and B1b cells, as previously described (50, 51, 54, 55). Anyhow, CD5 was expressed by a subset of B220^lo^ cells, while B220^hi^ cells were largely CD5 negative (**Figures 1B, C**). Most B220^hi^ cells expressed IgM in the lung and the spleen, whereas variable amounts of IgM were present in B220^lo^ cells (**Figure 1D**, upper panel; **Supplementary Figure 2A**). Similarly, IgD was highly expressed in adult B220^hi^ cells, but less in B220^hi^ cells at D7 and in all conditions of B220^lo^ cells (**Figure 1D**, middle panel; **Supplementary Figure 2A**). Hence, in lung and spleen significant numbers of B220^lo^ cells were negative for IgM and IgD (**Figure 1D**, bottom panel; **Supplementary Figure 2A**). The level of MHC-II was lower in cells from D7 mice compared to adults (**Supplementary Figure 2B**), as previously described for embryo B cells. Interestingly, at D7 the numbers of B cells displaying MHC-II were lower in lung B220^lo^ cells compared to B220^hi^ cells, whereas the reverse was observed in spleen.

The presence of IgM^-^IgD^-^ cells in B220^lo^ cells prompted us to prove markers identifying B cell precursors or immature cells. We used CD93, which is expressed from B cell precursors until the T2 stage in the spleen. We found that at D7 a high number of both B220^lo^ and B220^hi^ cells expressed CD93, whereas adult cells were negative (**Figure 1E**; **Supplementary Figure 2C**). Therefore, these IgM^+^CD93^+^ cells in lung and spleen may represent immature transitional B cells, which are abundant at D7 and not in adults.

We also checked for the presence of more differentiated cells among the B220^lo^ cell populations by the expression of the CD138 marker. We found that the D7 and adult spleens and adult lungs showed low number of CD138^+^B220^lo^ cells whereas B220^hi^ cells were CD138 negative (**Figure 1F**; **Supplementary Figure 2C**).

We analyzed the expression of CD43, CD9, and CD44 on B220^lo^ and B220^hi^ cells from D7 mice in comparison with B220^lo^ and B220^hi^ cells from adult tissues, and these with peritoneal B1 cells. CD43 expression has been shown to be present on conventional B1a cells and on B220^lo^CD11b^-^ cells in the adult spleen whereas CD9 and CD44 are also associated with B1-cell phenotype (51, 54, 55). Hence, high numbers of adult splenic and lung B220^lo^ cells expressed CD43, as did peritoneal B1a cells, and also B220^lo^ cells from lung and spleen at D7 (**Figure 1G**; **Supplementary Figure 2C, D**). In addition, we assessed the expressions of CD21 and CD23, which were, as expected, absent on peritoneal B1 cells and splenic B220^lo^ cells. However, approximately 6% to 10% of spleen and lung B220^lo^ cells were CD23^+^ (**Supplementary Figure 2D**). Likewise, B220^lo^ cells expressed CD9 (in both organs), although in this case the percentages of B220^lo^CD9^+^ cells were much higher at D7 than in adults. Finally, the pattern of expression of CD44^+^ cells was similar, although at higher numbers both at D7 and in adulthood (**Figure 1H**; **Supplementary Figure 2C**). In summary, lung B220^lo^ cells from D7 and adult lung share a B1-like phenotype with conventional B1 cells described in adult spleen and peritoneum.

### Microbiota modulation of B cell subpopulations

**Figure 2:**
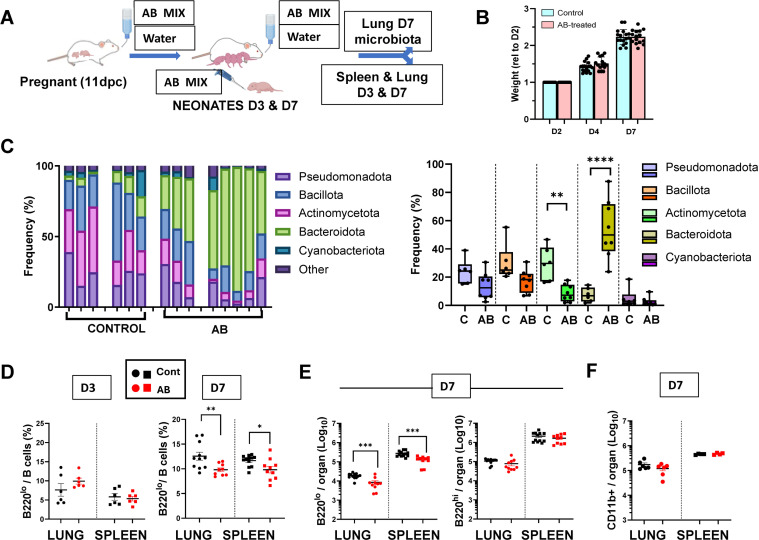
The lung microbiota affects the establishment of neonatal B lymphocytes. **(A)** Scheme of the antibiotic (AB mix) treatment of pregnant females and pups after delivery (from E11.5 to D3 or D7) and control mice receiving drinking water (Control), and the extraction of spleen and lung samples. **(B)** Effect of the antibiotic treatment on the weight of the pups is shown from D2, D4 and D7. The data are expressed relative to the one of each animal at D2. Data are presented as dots representing two independent experiments, each comprising two control and two antibiotic-treated litters (n = 17 control pups and 15 antibiotic-treated pups) The mean is shown by bars. **(C)** Quantification of the sequenced V4 16S rDNA from the lung samples from D7 litters exposed to antibiotics or not. Left, plots of microbiota species in lungs from individual mice. Right, frequency of Pseudomonadota, Bacillota, Actinomycetota, Bacteroidota and Cyanobacteriota in antibiotic treated or untreated lung samples. Data are plotted as box and whiskers graphs, with individual values as points, showing the median and the range values. **(D)** The effect of antibiotic treatment on D3 and D7 as the frequency of B220^lo^ over B cells in lung and spleen samples, quantified by flow cytometry, with samples stained with mAbs CD19-BV421 and B220-PE-Cy7. **(E)** Absolute numbers of B220^lo^ and B220^hi^ cells per organ from D7 lung and spleen samples treated or not with the antibiotic mix (n = 6-7). **(F)** Absolute numbers of CD11b^+^ cells per organ. The data in C-E are represented as dots, with the mean ± SEM indicated by lines (n = 6-7). The data were compared with One-way ANOVA with *post-hoc* Tukey correction: *P < 0.05; **P < 0.01; ***P < 0.001; ****P < 0.0001.

These results prompted us to test whether the microbiota might be involved in the early establishment and maintenance of the B220^lo^ cell population in the lung and spleen. We treated with a mix of oral antibiotics pregnant females from E11.5, as well as their pups from birth until their sacrifice (**Figure 2A**). In parallel, we analyzed age-matched litters from females that did not receive antibiotic treatment. We checked that the antibiotic treatment did not alter the weight of the pups, by weighing them at D2, D4 and D7 after birth (**Figure 2B**). We then verified the effect of the antibiotics on the microbiota by sequencing the V4 rDNA 16s in the lungs of the newborn pups at D7 and in the feces recovered from the cages. The lungs of antibiotic-treated newborns exhibited an altered bacterial composition compared with untreated littermates analyzed in parallel, characterized by increased Bacteroidota and reduced Actinomycetota, Pseudomonadota, and Bacillota (**Figure 2C**). Similarly, antibiotic treatment induced a compositional shift in fecal microbiota, characterized by reduced Bacteroidota and increased Pseudomonadota. (**Supplementary Figures 3A–C**). Additionally, albumin, glucose, and lactate dehydrogenase (LDH), were quantified in lung and intestinal samples obtained from D7 mice to evaluate tissue integrity (albumin and LDH) and metabolic status (glucose) (**Supplementary Figure 3D**). No differences were observed between antibiotic-treated and untreated neonates indicating preserved epithelial integrity without evidence of increased permeability or cytotoxicity, suggesting maintained metabolic homeostasis despite microbiota alteration. The effect of antibiotic-treatment on B220^lo^ and B220^hi^ cells was analyzed at D3 and D7. The antibiotic administration reduced the number of B220^lo^ cells in both the lung and spleen on D7 newborns, whereas not being changed at D3 (**Figures 2D, E**). Antibiotic treatment had a weaker effect on the number of B220^hi^ cells at D7 (**Figure 2E**) and had no effect on CD11b^+^ myeloid cells (**Figure 2F**). We also performed experiments in which oral antibiotics were either administered to the pups from birth until their sacrifice at D7 (**Supplementary Figure 3E**) or administered to pregnant females from embryonic day 11.5 (E11.5), with the offspring being culled at E18 and at D1 after delivery (**Supplementary Figure 3F**). Only in the first condition there was a reduction of B220^lo^ cells among B cells in the lung, as well as a reduction in absolute numbers of both lung and spleen samples. However, although these results suggest a direct effect of postnatal microbial exposure, we cannot discard indirect effects by alteration of immune cell development nor effects on other cell types (epithelial, myeloid) that provide survival signals.

**Figure 3:**
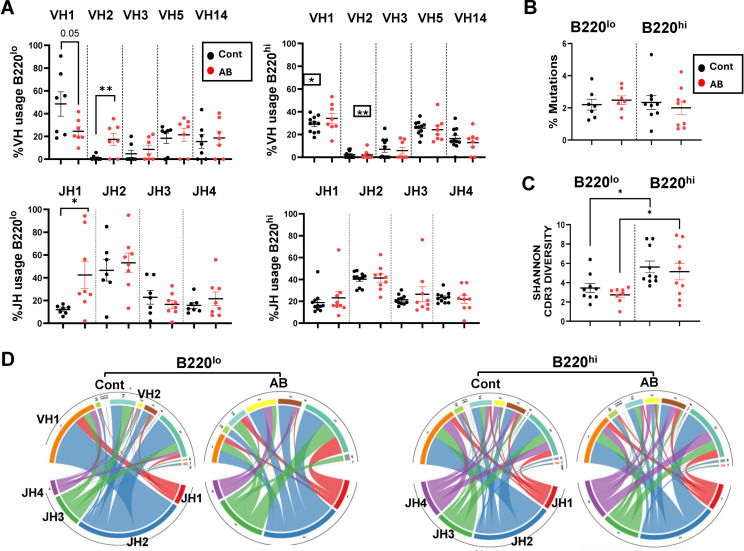
The lung microbiota affects the VDJCμ repertoires of neonatal B lymphocytes. Purified B220^lo^ and B220^hi^ pools were prepared by sorting splenic neonatal D7 cells treated with the antibiotic mix or not. RNA and cDNAs were prepared, and VDJCμ specific RT-PCR was performed, sequencing the amplified fragments and analyzing them on the IMGT bioinformatics platform. **(A)** The results of VH (upper panels) and JH (bottom panels) family usage in the VDJCμ rearrangements from purified B220^lo^ (left panel) and B220^hi^ (right panel) are shown (n = 7 for antibiotic-treated or untreated B220^lo^ and n = 9–11 for B220^hi^ antibiotic treated or untreated, respectively). The statistical significance values shown in the rectangles have been calculated by comparing VH1 and VH2 usage between B220^lo^ and B220^hi^ pools using One-way ANOVA with *post-hoc* Tukey correction. *P < 0.05; **P < 0.01. **(B)** The frequency of the mutations detected in the VH region of the VDJCμ sequences was obtained as detailed in Materials and Methods (n = 7 for B220^lo^ and n = 9 for B220^hi^, respectively). **(C)** Shannon CDR3 diversity is shown (n = 8 and 9 for B220^lo^ and 10 and 9 for B220^hi^ VDJCμ joints, treated or not with antibiotic. In all the graphs each dot represents a pool of five individual samples and the lines the mean ± SEM. The statistical analysis was performed using an unpaired Student’s *t*-test. *P < 0.05; **P < 0.01. Boxed stars in A represent the comparison of each group from B220^lo^ and B220^hi^ sequences. **(D)** Circos graphs of VH vs JH are shown for compiled samples of both B220^lo^ (190,000-200,000 sequences) and B220^hi^ cells (400,000-600,000 sequences) treated or not with antibiotics.

The effect of the antibiotics on the VH repertoires used by B220^lo^ and B220^hi^ cells was then analyzed. A total of 2,148,972 functional sequences were obtained by next generation sequencing of the VDJCμ fragments generated by PCR amplification from B220^lo^ and B220^hi^ sorted B cells from mice that received the antibiotics (327,241 and 443,682 sequences, respectively) or those that did not (269,092 and 1,108,957 sequences respectively, **Table 1**). Similar CDR3 length (as number of AA) were obtained for both populations in mice treated or not with antibiotics (**Table 1**). The VDJHμ repertoires were analyzed for each condition using pooled splenic B220^lo^ and B220^hi^ cells from D7 neonates, comprising eight and nine pools, respectively, from antibiotic-treated mice and seven and eleven pools, respectively, from untreated mice, with five individuals per pool in all cases. Interestingly, the antibiotic treatment provoked a change in the most commonly used VH families in the B220^lo^ cell repertoires, with a reduction in VH1 and increase in the VH2 and JH1 genes used (**Figure 3A**). By contrast, no changes were evident in the B220^hi^ cell repertoires. Antibiotic treatment did not alter the mutations in B220^lo^ cell nor B220^hi^ VH repertoires (**Figure 3B**), although, as found previously for adult samples (61), the B220^lo^ VH repertoires had less Shannon CDR3 diversity than those from the B220^hi^ cells (**Figure 3C**). Finally, circos plots illustrating VH-JH usage from pooled samples from control and antibiotic-treated B220^lo^ and B220^hi^ cells (**Figure 3D**) confirmed the changes on VH1, VH2 and JH1 gene usage observed in antibiotic-treated B220^lo^ cells.

**Table 1:** Summary of the sequences analysed in this study.

Sample	Treatment	Number of Sequences^1^	CDR3 length (AA)^2^
B220^hi^ #2	Control	83,192	10
B220^hi^ #14	Control	41,361	9
B220^hi^ #16	Control	20,814	10
B220^hi^ #22	Control	196,534	10
B220^hi^ #27	Control	185,386	10
B220^hi^ #24	Control	66,336	9
B220hi#2*	Control	95,878	9
B220hi#4*	Control	5,966	10
B220hi#10*	Control	17,295	10
B220hi#12*	Control	3,147	10
B220hi#20*	Control	393,048	11
Total		1,108,957	
Mean ± SEM			9.8 ± 0.2
B220^hi^ ·#6	Antibiotics	104,606	10
B220^hi^ #18	Antibiotics	10,408	12
B220^hi^ #20	Antibiotics	115,841	9
B220^hi^ #6*	Antibiotics	29,719	9
B220^hi^ #8*	Antibiotics	101,656	13
B220^hi^ #14*	Antibiotics	50,982	10
B220^hi^ #16*	Antibiotics	5,686	10
B220^hi^ #19*	Antibiotics	24,784	11
Total		443,682	
Mean ± SEM			11 ± 0.5
B220^lo^ #1	Control	51,781	9
B220^lo^ #13	Control	30,379	11
B220^lo^ #15	Control	23,751	10
B220^lo^ #23	Control	90,304	8
B220^lo^ #1*	Control	51,773	10
B220^lo^ #3*	Control	20,910	10
B220^lo^ #11*	Control	194	6
Total		269,092	
Mean ± SEM			9.1 ± 0.6
B220^lo^ #5	Antibiotics	82,023	9
B220^lo^ #17	Antibiotics	28,190	11
B220^lo^ #21	Antibiotics	10,834	9
B220^lo^ #25	Antibiotics	56,322	9
B220^lo^ #5*	Antibiotics	3,413	9
B220^lo^ #7*	Antibiotics	116,368	10
B220^lo^ #15*	Antibiotics	30,091	7
Total		327,241	
Mean ± SEM			9.1 ± 0.5
Total sequences analysed		2,148,972	

1 Pregnant females and neonatal mice were treated or not with antibiotics from E11.5 to the sacrifice as indicated in Methods. Purified splenic samples of B220^lo^ and B220^hi^ cells were obtained by preparative flow cytometry. After extraction of RNA and conversion to cDNA, specific VDHμ PCRs were performed as described in the Materials and Methods section, and the products were submitted to NGS. A summary of the sequences obtained is presented in the Table. ^1^Number of functional sequences obtained in each sample; ^2^CDR3 length as the number of aminoacids in the Complementary Determining Region 3 (CDR3).

### The absence of MyD88, TLR2 and TLR4 disrupts the B cell subpopulations

**Figure 4:**
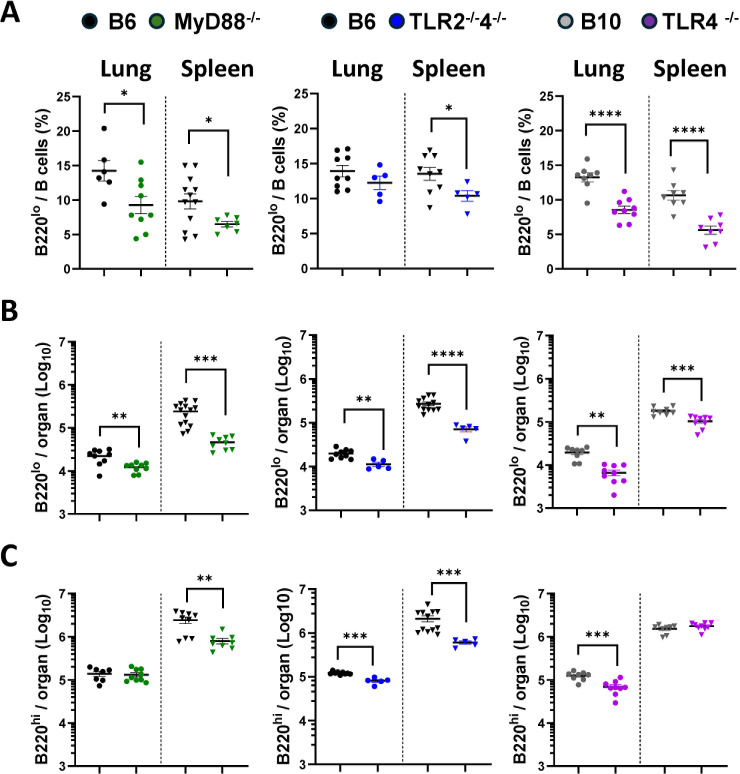
Low B220^lo^ cell counts in the absence of MyD88, TLR2 and TLR4 at D7. Cell suspensions were stained with mAbs CD19-BV421 and B220-PE-Cy7, The number of B220^lo^ cells from D7 in the lung (left, circles) and spleen (right, triangles) samples; BL6 mice in black, MyD88^-/-^ mice in green, TLR2^-/-^/TLR4^-/-^ mice in blue, BL10 mice in grey and TLR4^-/-^ mice in violet. **(A)** The results are expressed as relative to the B cells (% of B220^lo^/B cells); **(B)** as the absolute cell number/organ of B220^lo^ cells and **(C)** of B220^hi^ cells. The data are displayed as dots, each one representing an individual value (n = 5-14), plotting the mean as a horizontal line and the SEM as vertical lines, and comparing the data with One-way ANOVA with *post-hoc* Tukey correction: *P < 0.05; **P < 0.01; *** *P* < 0.001; ****P < 0.0001.

Since B cells use TLR receptors to sense the bacterial microbiota, the effect of the absence of TLR4, TLR4 and TLR2, and the MyD88 adaptor molecule on the B220^lo^ B cell numbers was assessed in deficient mice (**Figure 4**). MyD88-, TLR2/4- and TLR4-deficient mice had fewer B220^lo^ cells relative to B cells than WT mice at D7 in the lung and in the spleen (**Figure 4A**). Indeed, lung and spleen samples from D7 of the three deficient mice harbored less numbers of total B220^lo^ cells per organ (**Figure 4B**). A similar effect was found at D7 for B220^hi^ cells from TLR2/4 deficient mice, and from spleen and lung of MyD88^-/-^ and of TLR4^-/-^ mice, respectively (**Figure 4C**). B220^lo^ and B220^hi^ cells from adult samples from TLR2/4 deficient mice and B220^hi^ cells from TLR4^-/-^ mice were also diminished (**Supplementary Figure 4**). In summary, the absence of TLR2, TLR4 or MyD88 has a great effect from D7 on the number of B cells in lungs and spleen, affecting more profoundly the B220^lo^ compartment.

### Lung B cells respond to the TLR2 and TLR4 agonists *in vitro*

**Figure 5:**
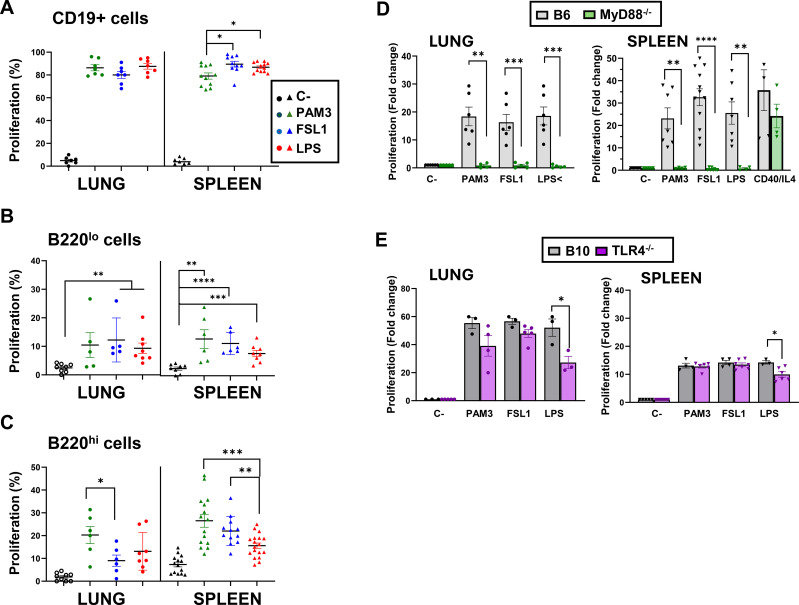
B cell responses after *in vitro* activation with TLR2 and TLR4 ligands. Lung and spleen cell suspensions from D7 BL6, MyD88^-/-^, BL10 and TLR4^-/-^ mice, and FACS-purified B220^lo^ and B220^hi^ from D7 BL6 mice were labeled with the violet dye at t = 0 and cultured in the presence of PBS **(C-)** or with the TLR2 (green PAM3, blue FSL1), TLR4 (red LPS) ligands for 72 hr, as detailed in the Material and Methods section, and then analyzed by flow cytometry. The cells were recovered and labeled with mAbs CD19-BV421 and B220-PE-Cy7. The frequency of the proliferating cells after one or more rounds of proliferation was determined by the decrease in the violet dye. **(A)** Proliferation of CD19^+^ cells (electronically gated) from BL6 mice lung (left, circles) and spleen (right, triangles) cultures. **(B)** Proliferation of purified B220^lo^ and **(C)** B220^hi^ cells from BL6 mice lung and spleen cultures. The individual data from 3 independent experiments are represented as dots with the mean ± SEM indicated as lines; n = 6–12 for CD19^+^ cells, n = 5–8 for B220^lo^ cells, and n = 6–18 for B220^hi^ cells. **(D)** Proliferation of electronically gated CD19^+^ cells from BL6 (light grey) and MyD88^-/-^ (green) mice. **(E)** Proliferation of electronically gated CD19^+^ cells from TLR4^-/-^ (violet) and BL10 (dark grey) mice. The bars in D and E represent the fold change relative to the proliferation induced by the medium alone. The mean ± SEM is displayed as bars, with dots representing the individual data (n = 6–12 for BL6, n = 5–8 for MyD88-/-, n = 3–5 for BL10 and n = 4–7 for TLR4-/-). The data was compared using One-way ANOVA with *post-hoc* Tukey correction: *P < 0.05; **P < 0.01; *** *P* < 0.001; ****P < 0.0001.

The effect of TLR agonists on the maintenance and activation of D7 B cells *in vitro* was then tested, using PAM3, FSL1 and LPS as TLR2/TLR1, TLR2/TLR6 and TLR4 agonists, respectively, to stimulate lung and spleen cells. The effect of these ligands was analyzed after 3 days in culture. When stimulated with any of the agonists, the proliferation of CD19^+^ B cells was much higher than those incubated with the medium alone (**Figure 5A**). To test the direct effect of the agonists over B220^lo^ and B220^hi^ cells, the cultures were performed using FACS-purified cells (**Figures 5B, C**). In both cases, the increase in proliferation was much lower than that obtained with unsorted cells, and B220^lo^ cells exhibited markedly less proliferation compared to B220^hi^ cells in spleen cultures (*P < 0.001*). These last cells proliferated less when purified from lungs, particularly after activation with FSL1, and LPS was the agonist that induced less proliferation in splenic B220^hi^ cell cultures (**Figure 5C**).

These results were validated using cells from MyD88^-/-^ and from TLR4^-/-^ mice. Cells from MyD88^-/-^ mice were unable to survive or proliferate when the cultures were stimulated with TLR agonists, whereas they did respond to anti-CD40+IL4 stimulation (**Figures 5D**, right). By contrast, TLR4^-/-^ cells responded to TLR2 agonists but much less to LPS, as expected (**Figure 5E**). Therefore, B cells present at D7 in the lung and spleen can survive and proliferate when stimulated through TLR2 and TLR4 signaling pathways.

### Lung B cells differentiate to plasma cells and secrete immunoglobulins in response to TLR agonists *in vitro*

**Figure 6:**
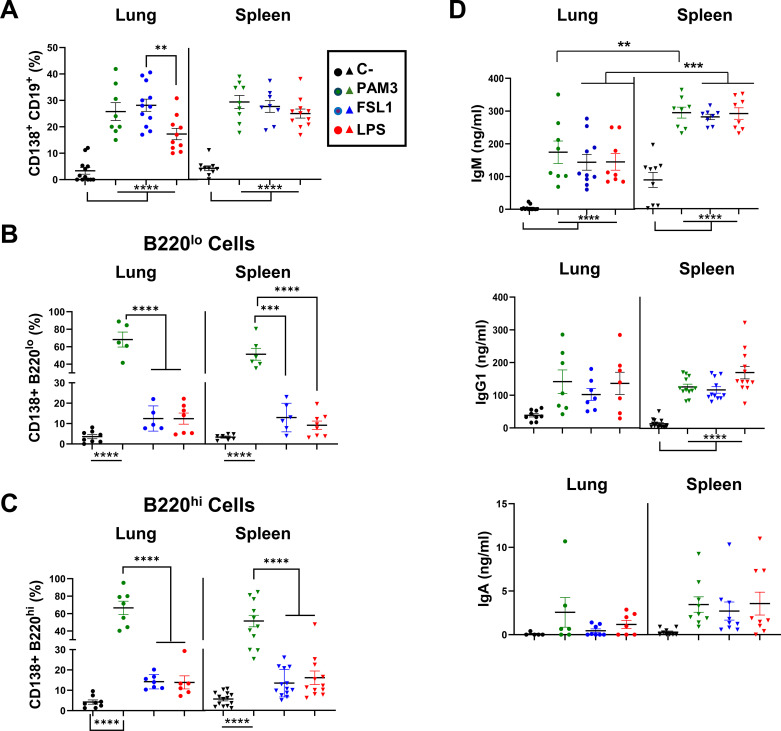
* In vitro* differentiation of neonatal B cells after activation with TLR2 and TLR4 ligands. Cell suspensions from lungs and spleens and from FACS-purified B220^lo^ and B220^hi^ cells of D7 BL6 mice were cultured for 72 hr in the presence of PBS (C-,black), PAMP3 (green), FSL1 (blue) and LPS (red), as described in **Figure 5**, and then the cells and the supernatants were recovered. The cells were stained with mAbs against CD19-BV421, B220-PE-Cy7 and CD138-APC, and analyzed by flow cytometry. The supernatants were stored at -20 °C until the immunoglobulins and cytokines were measured. **(A)** Frequency of the CD138^+^CD19^+^ cells in cultures from cell suspensions of lung (left, circles), and in spleen (right, triangles). n = 8–12 in lung cell cultures and n = 8–11 in splenic cultures. **(B)** Frequency of CD138^+^B220^lo^ in cultures of FACS-purified B220^lo^ lung cells (left, circles), and B220^lo^ spleen cells (right, triangles). n = 5–8 in lung cell cultures and n = 6–8 in splenic cultures. **(C)** Frequency of CD138^+^B220^hi^ in cultures of FACS-purified B220^hi^ lung cells (left, circles), and B220^hi^ spleen cells (right, triangles). n = 6–8 in lung cell cultures and n = 11–14 in splenic cultures. **(D)** Quantification of immunoglobulins from lung (left, circles) and spleen (right, triangles) cultures in supernatants analyzed for IgM (upper graph), IgG1 (middle graph) and IgA (bottom graph) by ELISA. The individual data from 3 independent experiments are represented as dots, with the mean ± SEM indicated as lines. Lung IgM n = 8-12, IgG1 n = 7-9, IgA n = 5-8; Spleen IgM n = 8-9, IgG1 n = 11-14, IgA n = 9). The comparisons were determined using One-way ANOVA with *post-hoc* Tukey correction: *P < 0.05; **P < 0.01; *** *P* < 0.001; ****P < 0.0001.

The effect of TLR2 and TLR4 agonists on the differentiation of D7 B cells *in vitro* was then tested after stimulation of lung and spleen cells, and of FACS-purified B220^lo^ and B220^hi^ cells. The effect of these ligands was analyzed after 3 days cultures as their differentiation to CD138^+^ B cells. There were more CD138^+^ B cells in the lung cell cultures after TLR2 stimulation than when stimulated with LPS (**Figure 6A**, left), whereas the three stimuli produced similar numbers of these cells in the spleen cultures (**Figure 6A**, right). By contrast, the cultures performed with FACS-purified B220^lo^ cells (**Figure 6B**) and with B220^hi^ cells (**Figure 6C**) showed a different pattern of response: Although there was an induction of CD138^+^ cells with the three agonists, it was much higher with PAM3 in either of the B220^lo^ and B220^hi^ cells lung and spleen cultures. These results suggest that isolated B cells need the presence of other cells or soluble mediators to reach a high response to TLR2 and TLR4 agonists and differentiate better through the TLR2/TLR1 stimulation with PAMP3 than to TLR2/TLR6 or TLR4 activation.

When IgM, IgG1 and IgA were quantified in the cultures, there were no differences for IgM among the agonists (**Figure 6D**, top), although more IgM was secreted from spleen cultures than lung cultures. IgG1 production was induced by all three agonists in both lung and spleen cell cultures; however, LPS stimulation elicited higher IgG1 levels in spleen cells than PAM3 or FSL1 (**Figure 6D****, middle**). Finally, IgA was also detected at low levels in supernatants from spleen cells even at 3 days in culture (**Figures 6D**, bottom).

### Cytokine secretion in lung and spleen cultures is activated by TLR2 and TLR4 agonists

**Figure 7:**
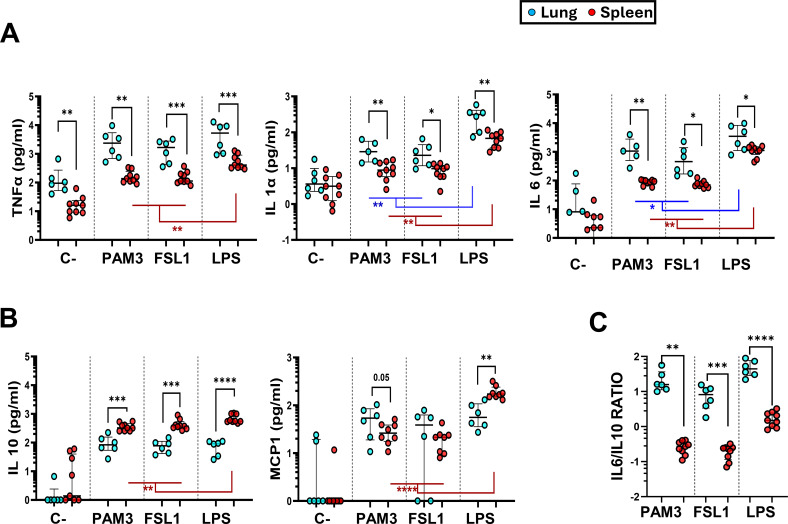
Cytokine quantification from D7 lung and spleen cultures activated with TLR2 and TLR4 ligands. D7 cells from the lung and spleen were cultured in the presence of PAM3, FSL1 and LPS for 72 hr, as indicated in **Figure 4** and **5**. The cytokines in the supernatants were detected by the cytometric bead array assay. **(A)** The results for TNF-α, IL1-α and IL6 in lung (blue dots) and spleen (red dots) culture supernatants (n = 5–6 for lung samples and (n = 9 for spleen samples). **(B)** The results for IL10 and MCP1 are shown as in A **(C)** Comparison of the IL6/IL10 ratio between lung and spleen cultures (n = 6-9). The individual data are displayed as dots, and the mean ± SE as horizontal and vertical lines, respectively. Significance was determined with One-way ANOVA with *post-hoc* Tukey correction: *P < 0.05; **P < 0.01; *** *P* < 0.001; ****P < 0.0001.

When the cytokine content in the supernatant of the cultures was analyzed (**Figure 7**), there was an induction of TNFα, IL1α, IL6, IL10 and MCP1. Lung cultures had less IL1α and IL6 proinflammatory cytokines after FSL1 activation than when stimulated with LPS (**Figure 7A**). Moreover, cultures of spleen cells stimulated with PAM3 or FSL1 had less TNFα, IL1α, IL6, IL10 and MCP1 than those activated with LPS (**Figure 7**). Interestingly, cytokine secretion profiles differed markedly between organs: lung cultures produced higher levels of TNFα, IL1α, and IL6 compared to spleen cultures (**Figure 7A**), whereas spleen cells secreted more IL10 and MCP1/CCL2 (the last in response to LPS) (**Figure 7B**). Hence, the IL6/IL10 ratios were much higher in lung cultures than in spleen cultures (**Figure 7C**).

**Figure 8:**
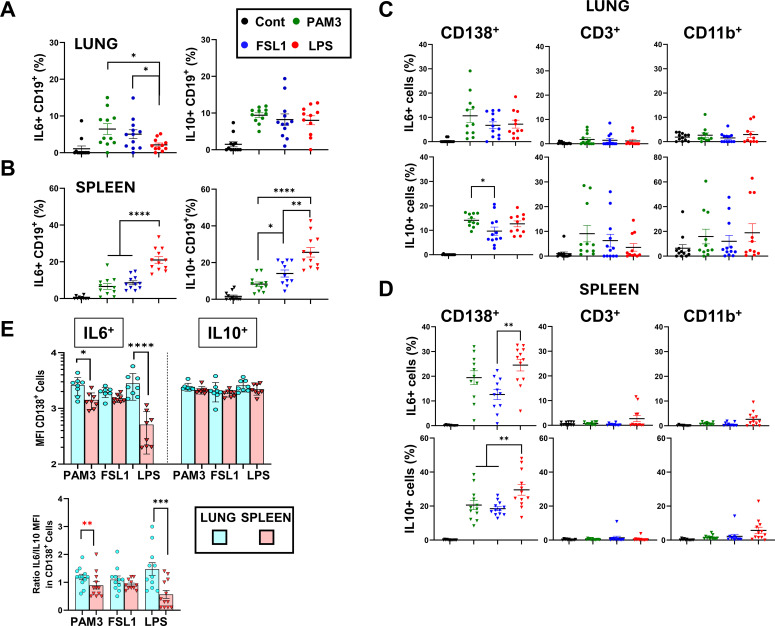
Quantification of intracellular production of IL6 and IL10 by CD19^+^ cells growing in D7 lung and spleen cultures activated with TLR2 and TLR4 ligands. At the end of the cultures (D3), the intracellular production of IL6 and IL10 was determined by flow cytometry by using anti-CD19-BV421, anti-B220-PE-Cy7, anti-CD138-APC, anti-CD3_FITC, anti-CD11b-APC-Cy7, anti IL6-PE or anti-IL10-PE MoAbs. **(A)** CD19^+^ cells producing IL6 or IL10 in lung cultures; left, IL6^+^CD19^+^ cells; right, IL10^+^CD19^+^ cells (n = 9-12). **(B)** CD19^+^ cells producing IL6 or IL10 in spleen cultures; left, IL6^+^CD19^+^ cells; right, IL10^+^CD19^+^ cells (n = 12-13). Results in A and B are presented as dot plots, in which each dot represents the result of an individual culture; the mean ± SE shown as horizontal and vertical lines, respectively. **(C)** CD138^+^ cells (plasmablasts, left), CD3^+^ cells (T cells, middle) and CD11b^+^ myeloid cells (right) producing IL6 (upper graphs) or IL10 (bottom graphs) in lung cultures; (n = 11-12). **(D)** Data are as in C, from cultures of spleen cell suspensions; (n = 11-12). **(E)** Top: Comparison of the MFI of IL6 and IL10 displayed in the CD138^+^IL^+^ cells. Bottom: Ratio of IL6/IL10 MFI in CD138^+^ cells. Data are individual values displayed as dots and the mean ± SEM (n = 11-15). Significances were determined with one-way ANOVA test with Tukey correction (black) or unpaired t-Student (red): *P < 0.05; **P < 0.01; ****P < 0.001.

In terms of cells secreting IL6 and IL10, intracellular staining after 3 days of stimulation indicated that most IL6^+^ or IL10^+^ cells were B cells (**Figure 8A** for lung, **Figure 8B** for spleen; **Supplementary Figure 5A**), and also there were more IL6^+^ and IL10^+^ cells in the CD138^+^ cell population than in the CD3^+^ T cells or CD11b^+^ myeloid cells, both in the stimulated cultures of the spleen and lung (**Figure 8C** for lung, **Figure 8D** for spleen).

**Figure 9:**
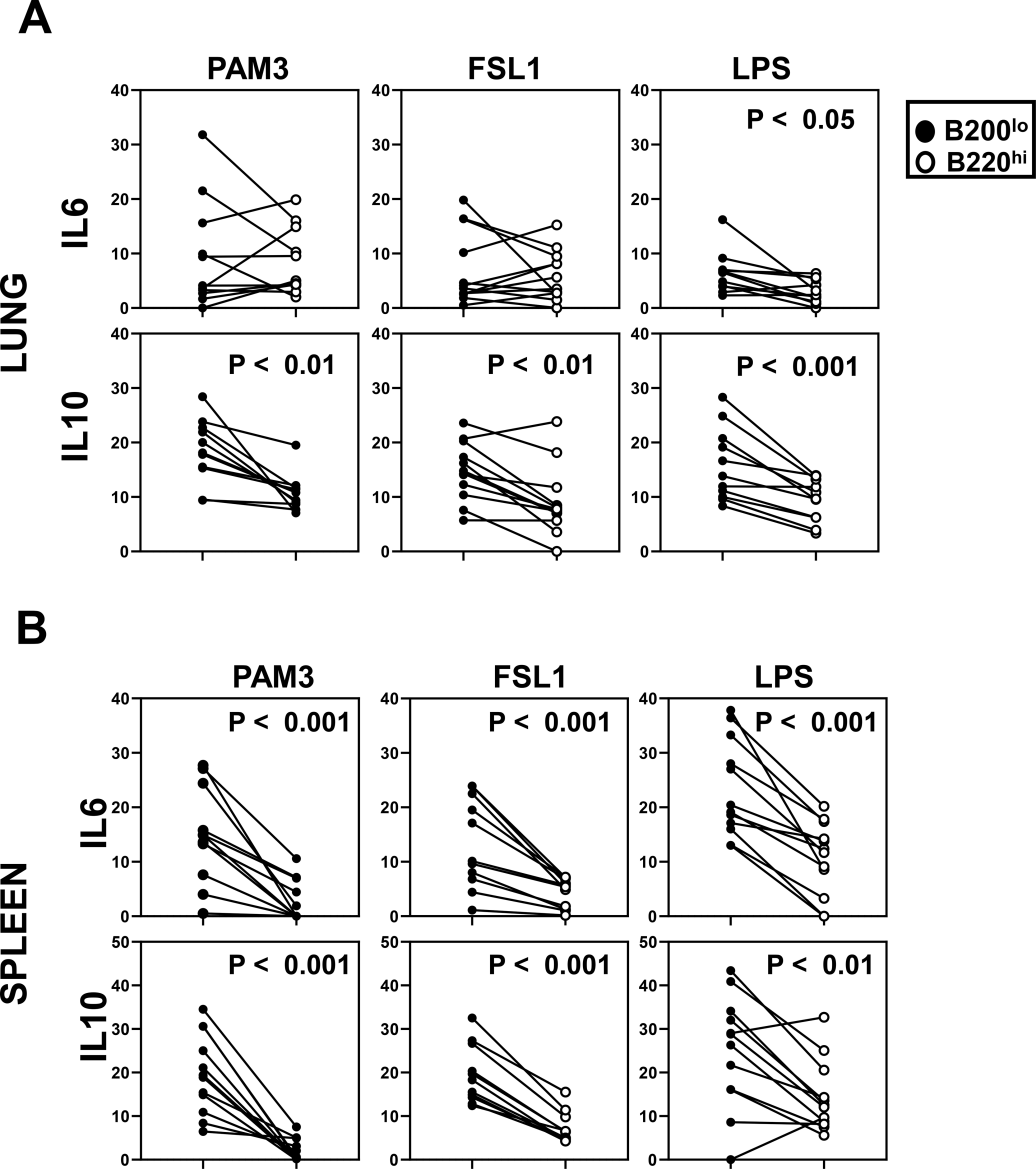
Comparison of IL6 and IL10 producing B220^lo^ and B220^hi^ cells growing in the same cultures. Cells were stained at the end of the cultures as indicated in **Figure 8**. **(A)** Data are displayed as pairwise graphs. The results for B220^lo^ (filled) and B220^hi^ (empty) are represented by dots, and the connecting lines identify each pair of cells obtained for the same culture of lung cell suspensions. The numbers inside are the significances, obtained with One-way ANOVA with *post-hoc* Tukey correction; n = 11-12. **(B)** Data are as in E, from cultures of spleen cell suspensions.

When IL6^+^ and IL10^+^ B cells were compared in the lung and spleen cultures (**Supplementary Figure 5B**), there were more of these cells in TLR2/4-activated spleen cultures. By contrast, the mean fluorescence intensity (MFI) of IL6 was higher in CD138^+^ cells from TLR-stimulated lung cultures (**Figure 8E**), whereas the IL10 MFI was similar in all conditions. Therefore, the ratio of IL6 MFI to IL10 MFI among CD138^+^ cells from lung and spleen cultures was higher in lung cells (**Figure 8E**). This may indicate that lung CD138^+^ B cells could secrete more IL6 than splenic B cells, contributing to the higher IL6 levels in the supernatants of lung cultures. When analyzed separately in function of B220 surface expression, more IL6^+^ and IL10^+^ cells were B220^lo^ than B220^hi^ cells in the lung and spleen cultures (**Figure 9A** for lung, **Figure 9B** for spleen).

### Lung B cells respond to TLR2 agonists *in vivo*

**Figure 10:**
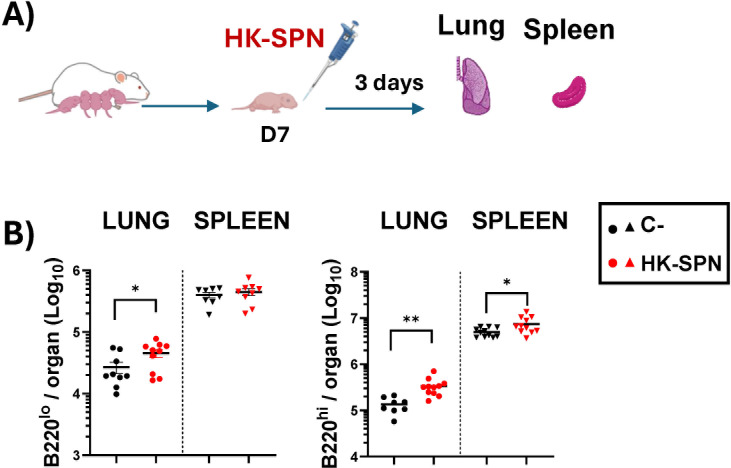
*In vivo* response to Heat Killed-SPN (HK-SPN). **(A)** BL6 mice were immunized via intranasal at postnatal D7 with the TLR2 agonist HK-SPN, as indicated in the Methods section. **(B)** The mice were sacrificed at D10, and the B220^lo^ and the B220^hi^ cells present in the lung (dots) and in spleen (triangles) samples were assessed in the control (black) and immunized mice (red). The data are presented as dots for individual values and the mean ± SEM; n = 9-12. Upper panels, absolute numbers per organ of B220^lo^ (left graph) and B220^hi^ cells (right graph). Comparisons were assessed with One-way ANOVA with *post-hoc* Tukey correction: *P < 0.05; **P < 0.01; ***P>0.001.

We administered intranasally HK-SPN as an *in vivo* TLR2 agonist to D7 BL6 mice and analyzed its effect on the lung and spleen B cells (**Figure 10A**). Three days later there was an increase in the total number of B220^lo^ and B220^hi^ cells in the lung and B220^hi^ cells in spleen of BL6 mice (**Figure 10B**).

## Discussion

The role of TLRs during pregnancy and after delivery has been attributed to immune responses against the placental microbiome, tissue morphogenesis and a response to short-chain fatty acids (72). Transitioning from fetal to perinatal life involves the simultaneous development of immune responses and the microbiome. Colonization of secondary lymphoid organs by immature immune cells occurs at the end of the gestation and perinatally (73, 74), the latter a period in which the IS and the surrounding microbiome mature concomitantly (75). The skin, gut and lungs are the sites where epithelial cells come into contact with the microbiome and pathogens (76), and consequently they harbor the cells of the IS that are involved in its first response, then recruiting more myeloid and lymphoid cells. The lung starts to develop at 3 weeks of human pregnancy and at E9.5 in mouse embryo (77). Dendritic cells and embryonic macrophages are the first immune cell populations to colonize the lung (78), and their initial functions are mainly related to tissue remodeling and airway branching (79), whereas NK cells in the embryonic lung are more closely involved in cytokine production (80).

We previously identified a novel population of B220^lo^CD11b^-^ B lymphocytes of embryonic origin that is closely related to the B1 lineage, as are splenic B1 cells (50, 51). These cells express markers of activation and differentiation (i.e. CD43, CD138), display intrinsic activation/proliferative state, spontaneous IgM secretion and predominant embryonic origin (54, 55). These characteristics distinguish them from conventional B2 cells and align them with an innate-like B-cell program, regardless of their phenotypic divergence from peritoneal B1 cells, also based on their cell cycle cascade program, their ability to secrete IL10 upon *in vitro* activation and harbor an unexpected capacity to secrete IgG and IgA antibodies (54, 55). Thus, studying B220^lo^ cells in the neonatal lung will provide insights into how early-life B-cell populations are established within barrier tissues and how they may shape local immune responses from birth onward. By tracing these B cells during neonatal aging, we demonstrate that these B220^lo^ cells are enriched in the first 7 days after birth in the lung and spleen, with a similar phenotype than the adult splenic B220^lo^ cells, except for CD138 in the lung.

*In vivo* treatment of pregnant females and of neonates after birth with a combination of antibiotics did not affect neither epithelial integrity nor inflammation-associated biochemical parameters and maintained metabolic homeostasis, despite effectively altered the bacterial microbiome. In fact, the effect was mostly due to the treatment of pups in early life after delivery. This approach led to fewer post-natal B220^lo^ cells at D7 in the lung and spleen, whereas the number of perinatal CD19^-^CD11b^+^ cells does not change after antibiotic treatment. Furthermore, the alteration of the microbiome has more specific impact on the neonatal repertoire of B220^lo^ than of B220^hi^ cells. It is conceivable that B220^lo^ cells require positive selection signals from specific self or microbial antigens to survive and proliferate in the periphery, as occurs with conventional B1 cells, thus being highly susceptible to pulmonary microbiota and antibiotic induced dysbiosis. These results highlight the relevance of antibiotic exposure during infancy and the influence this has on B cells, which may affect future immune responses in the adult.

To assess the influence of TLRs on the establishment of perinatal B lymphocytes, we studied deficient MyD88^-/-^, TLR4^-/-^, and double TLR2^-/-^/TLR4^-/-^ mice under homeostatic conditions. At D7, the proportion of B220^lo^ cells in the B cell population of the lung and spleen was reduced in the absence of MyD88, TLR4 and both TLR2 and TLR4. By contrast, adult MyD88^-/-^ mice recovered B220^lo^ B cell numbers, reaching higher levels in spleen compared to that from BL6 controls (**Supplementary Figure 3**), whereas adult samples from TLR2/4-deficient mice remained diminished. It was described that there are more MZB cells and CD1d^hi^CD5^+^ cells in the spleen of adult MyD88^-/-^ mice (81), like we have found here for B220^lo^ B cells. Preferential bacterial sensing by TLR2 but not TLR4 has been demonstrated in D7 neonatal mice after Chlamydia respiratory infection (82). Neonatal TLR2^-/-^ infected mice develop more severe infections and stronger inflammation than control mice (more NK cells, neutrophils, dendritic cells and activated CD8^+^ T cell influx and IL17 responses), whereas infections were milder in TLR4^-/-^ mice (82).

We studied *in vitro* cultures of perinatal lung and spleen cells stimulated with TLR2 and TLR4 ligands to assess the effects of TLR stimulation on perinatal B cell proliferation/differentiation pathways. As occurs with LPS, the TLR2/TLR6 and TLR2/TLR1 ligands induce CD19^+^ cell proliferation, differentiation to CD138^+^ cells, the production of IgM, IgG and IgA, and the secretion of proinflammatory and regulatory cytokines. The proliferation and differentiation of purified B220^lo^ and B220^hi^ in D7 cultures was lower than in those performed with complete cell suspensions although the numbers of B220^lo^ and B220^hi^ cells were adjusted to provide cell contacts. Our previous results with purified adult splenic B220^lo^ cells showed that they were responsive to LPS, CpG. BAFF/APRIL+IL4 and anto-CD40+IL4 (54, 55). The low proliferation of D7 purified cells stimulated with TLR agonists found here may be due either to the lack of myeloid or epithelial cells, together with the absence of soluble mediators in the purified preparations, or it may happen that neonatal B cells may need stronger activation signals to induce the proliferation/differentiation programs. The first interpretation is supported by our preliminary data showing an increase in proliferation from lung B220^hi^ cell cultures supplemented with cells from autologous purified non-B cells added back to the culture (*unpublished results*). Additional studies are required to fully elucidate the contribution of accessory cells to neonatal B-cell proliferation and differentiation, as well as to identify the cellular sources and soluble mediators involved.

Cytokines such as IL-1 and GM-CSF are likely contributors, as IL-1 enhances B-cell entry into the cell cycle and responsiveness to growth signals (83), whereas GM-CSF indirectly promotes B-cell activation by stimulating myeloid cells to produce B-cell-supporting cytokines. These mechanisms amplify B-cell responses to TLR stimulation and account for the enhanced activation observed in mixed cell cultures compared with purified B-cell cultures (84, 85).

Finally, the TLR2/TLR1-agonist PAMP3 acting over the purified B220^lo^ and B220^hi^ cells induced much higher differentiation to CD138^+^ cells than TLR2/TLR6 and TLR4 agonists.

In humans, the IL6/IL10 ratio has been used as a marker of neonatal sepsis that may predispose the organism to a worse prognosis after treatment (86). Our data show a higher IL6/IL10 inflammatory ratio in supernatants from lung cultures relative to spleen cultures after stimulation with TLR2 and TLR4 ligands. Furthermore, B cells, and particularly CD138^+^ pre-plasma/plasma cells, are responsible for most of the secretion of these cytokines, with a small contribution of other cell types present in the cultures. Indeed, the data suggests that lung B220^lo^ cells and CD138^+^ cells produce more IL6 than IL10 after three days of stimulation. MCP1 is implicated in different pathologies, promoting the homing of monocytes to target organs in response to inflammation (87). In our cultures, MCP1 was only weakly induced in lung and spleen cultures exposed to TLR2 agonists, and only LPS enhanced MCP1 secretion in spleen cultures. Although we did not determined the precise cell type that secretes this cytokine, the data indicates that neonatal spleen may respond to TLR4 stimulation by recruiting monocytes to drive the inflammatory response (88).

As expected, neonatal TLR4^-/-^ cells in culture respond to TLR2 ligands but not to LPS by increasing their proliferation and viability. Likewise, cultured neonatal MyD88^-/-^ cells were unable to respond to TLR2 and TLR4 ligands, confirming that neonatal CD19^+^ cells were able to transduce TLR2/TLR4-MyD88 dependent signals. When the *in vivo* response to SPN was assessed on neonatal mice, we obtained a high mortality rate in mice that were infected at D14 (**Supplementary Figure 6A**). However, when the infection was performed in adult mice there was an increase in lung CD19 cell numbers (**Supplementary Figure 6B**). Furthermore, *in vivo* immunization of D7 BL6 mice by instillation of HK-SPN produces a specific effect on WT B cells, showing that B220^lo^ cells can respond to TLR2 agonists in these conditions. These results are consistent with the finding that peripheral blood cells from human newborns respond to Gram^+^ bacterial infection *in vivo* by amplifying the TLR2-MyD88 pathway, whereas Gram^-^ bacterial infection amplifies the TLR4/MD2/MyD88 pathway (15, 89). However, the contribution of B cells to the control of neonatal infections remains to be clarified, due to the immature phenotype of these cells, and their small numbers in the blood and peripheral organs. Our findings demonstrate the potential of TLR-dependent activation and differentiation of certain neonatal B cell populations with an innate B1 phenotype, which further underlines the relevance of early antigen/microbiota encounters in shaping the immune B cell compartment, and particularly the innate-like B cell populations.

Under homeostatic conditions B1 cells are found in different non-lymphoid organs like the lung (90) and although they share several phenotypic traits with the B220^lo^ cells identified here as both being B220^lo^IgM^+^CD43^+^CD21^-^, they differ in their CD11b expression (positive in all peritoneal B1 cells) and in the variable expression of CD5 by B220^lo^ cells, which points to an heterogeneous composition of this cell subset in the post-natal lung. Adult splenic B220^lo^ cells share with peritoneal B1 cells intrinsic activation/proliferative state, spontaneous IgM secretion and predominant embryonic origin (54, 55). These characteristics distinguish them from conventional B2 cells and align them with an innate-like B-cell program, regardless of a divergence from peritoneal B1 cells based on their cell cycle cascade program, their ability to secrete IL10 upon *in vitro* activation, but making them close to B1a cell immigrants in spleen. B1a cells are CD5^+^ and produce natural IgM antibodies, important for early protection (91), while B1b cells are CD5^-^ and they contribute substantially to adaptive antibody responses against *S. pneumoniae*, producing protective antibodies against capsular polysaccharides after pneumococcal immunization (92). Down-regulation of CD5 was demonstrated after TLR activation of B1a CD5^+^ cells, and after influenza virus and *Salmonella typhimurium* infection (93).

**Figure 11:**
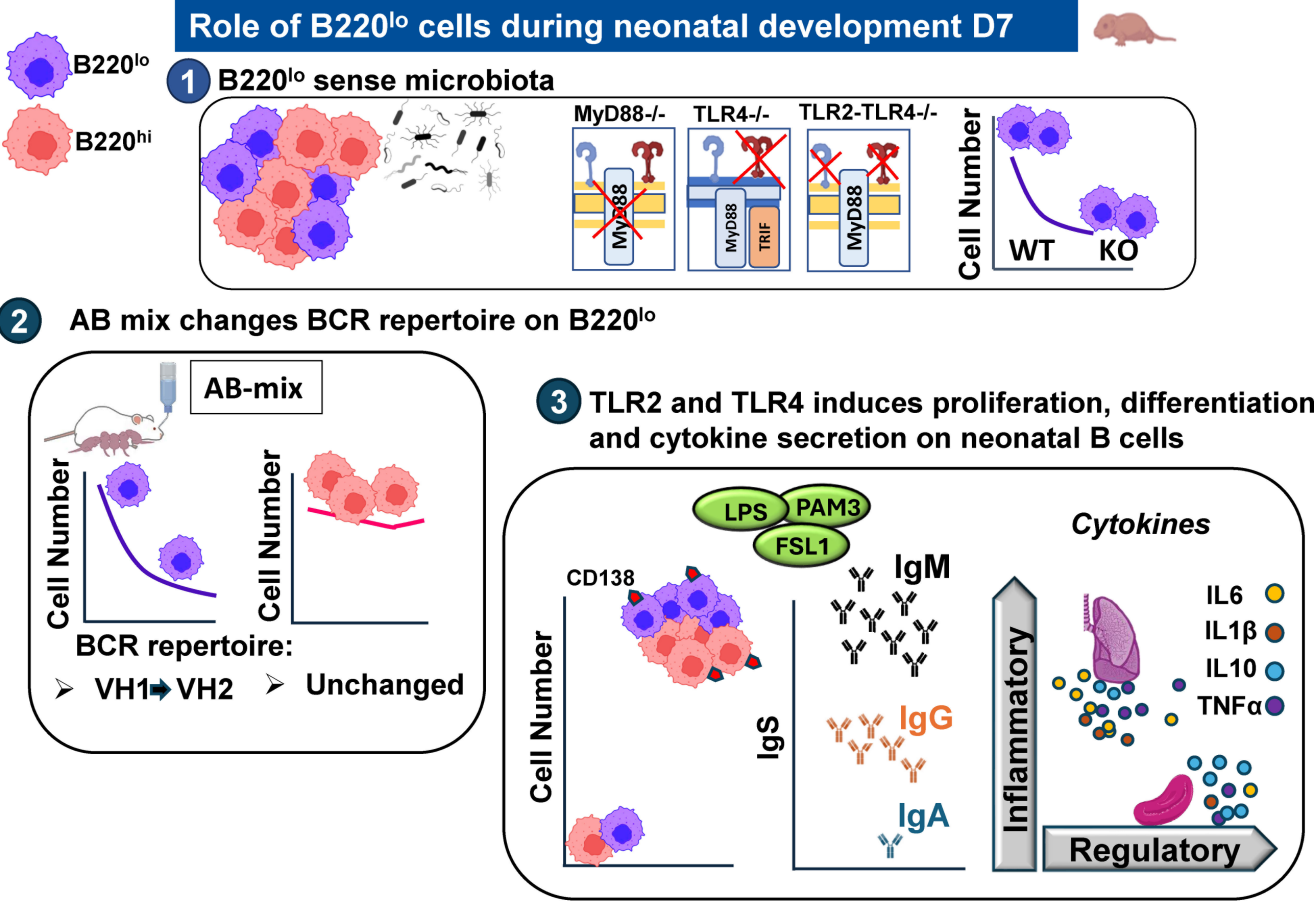
Graphical summary of the major findings.

All these findings led us to conclude (**Figure 11**) that the establishment of perinatal B cells depends at least on the tonic signals mediated by TLR2 and TLR4, and the maternal microbiome. In this sense, TLR2/4 had more evident effects on B220^lo^ than on B220^hi^ cells, suggesting an active role of the former compartment in the perinatal lung and spleen. Furthermore, the secretion of cytokines by B220^lo^ and CD138^+^ cells strengthens their role as regulators of immune responses during early postnatal life, in conjunction with other embryonic cells such as lung NK cells (80).

## Data Availability

The datasets analyzed for this study can be found in the European Nucleotide Archive: accession number: PRJEB109791.
