# Supplementary material for: Neonatal CD19+B220lo cells sense microbiota via TLR2/4 activation driving proliferation and differentiation
Source: Front Immunol. 2026 Jun 19;17:1838044. doi: 10.3389/fimmu.2026.1838044 (PMC13327984; doi:10.3389/fimmu.2026.1838044)
Supplement: Supplementary file 1 [file DataSheet1.pdf]

Supplementary Material

Neonatal CD19^{+}B220^{lo} cells sense microbiota via TLR2/4 activation driving proliferation and differentiation

C. Ruiz-Sánchez, I. Cortegano, M. Rodríguez, R. Sánchez-Tarjuelo, A. Arrabal, C. Prado-Zamora, M. Alía, P. Jiménez, V.M. López, S. Monzón, V. López-Alonso,

B. de Andrés*, M.L. Gaspar*

Correspondence: Belén de Andrés or Maria Luisa Gaspar (ORCID: 0000-0001-9858-3862): bdandres@isciii.es; mlgaspar@isciii.es.

Supplemental Material

Supplementary Methods

Analysis of Biochemical parameters in lung and intestinal homogenized tissues

Albumin, LDH and glucose levels were measured using an automated clinical chemistry analyzer (SPIN200E, Spinreact, Spain), following the manufacturer's instructions. A total volume of 70 µL per sample was used for each determination. Samples analyzed included supernatants from neonatal lung and intestinal homogenates collected at D7, treated or untreated with antibiotics, as well as control serum samples from D7.

Infection of neonatal and adult BL6 mice with *Streptococcus pneumoniae* (SPN)

Intranasal infection of D14 and adult BL6 mice with SPN was performed as in reference 16. D14 mice were infected with different doses of SPN (1×10^5–1×10^6 CFU). Survival was monitored every day after infection. Adult C57BL/6 and C57BL/10 mice were infected with 2×10^6 CFU of SPN. At day 3 post-infection, lung and spleen samples were processed and stained with anti-CD19 before flow cytometry procedures.

Legends to Supplemental Figures

Supplementary Figure 1: Schematic representation of the analyses performed. (A) The representative cytometry plots from a lung sample obtained from D7 BL6 mice are shown. Electronically gated CD19+ cells were separated as B220^{lo} and B220^{hi}, and the expression of several markers were then shown in these populations. The histograms on the right show an example of this analysis.**(B)** Scheme of the cell cultures performed with lung cell cultures, including representative dot plots and histograms determined by flow cytometry with cells

obtained from lung cultures after a 3 day stimulation with TLR2 or TLR4 agonists. The B220 phenotype of the $CD19^+$ B cells growing in the cultures is shown: proliferation (as determined by the decrease in the violet cell dye) and differentiation (indicated by the appearance of $CD138^+$ B cells). The number of IL6 or $IL10^+$ B cells was determined by intracellular flow cytometry. The Igs and cytokines secreted in the cultures were determined in the supernatants by ELISA and CBA assays, respectively.

Supplementary Figure 2: Extended phenotype of the neonatal B lymphocytes. Cell suspensions of lung and spleens were stained with mAbs against CD19-BV395, B220-APC, CD5-AF647, CD11b-PE-Cy7, IgM-FITC, IgD-BV605, or with CD19-BV421, B220-PECy7, MHC-II-FITC, CD93-APC, CD138-BV711, CD44-BV510 and CD43-PE or CD9-PE, and were thereafter analyzed by flow cytometry on electronically gated $B220^{lo}$ and $B220^{hi}$ cells, excluding dead cells with the live-dead reagent BV510 or with PI. **(A)** Representative overlayed contour plots displaying (in red) the IgM vs IgD profile of $B220^{lo}$ (top) and $B220^{hi}$ (bottom) of lung and spleen cells from D7 mice. The staining of $CD11b^+$ cells is shown in blue. The bottom graphs show the quantification of the percentages of cells defined by the quadrants as the mean ± SEM, and each value is displayed as a dot. n = 9-12 for D7 samples and n = 6-7 for adult samples. **(B)** The bar plot represents the MFI of cells positive for IgM on electronically gated cells as in A. The bars are the mean ± SEM, n = 5-6. The data were compared with an unpaired Student's *t*-test: $^{**}P < 0.01$; $^{****}P < 0.0001$. **(C)** Representative cytometry histograms on electronically gated $B220^{lo}$ and $B220^{hi}$ cells of D7 and adult lung and spleen cell populations stained with the indicated mAbs. Vertical lines show the value of the staining of negative populations. (D) Comparison of the expression of CD43, CD21, CD23 and CD11b between peritoneal B1a and B1b cells and lung and splenic $B220^{lo}$ cells. Left plots show the staining used for B1a and B1b selection by electronic gating. Middle plots show representative cytometry histograms on electronically gated adult peritoneal B1a and B1b cells, and adult lung and spleen $B220^{lo}$ and $B220^{hi}$ cells populations stained with the indicated mAbs. Right, the bar plot represents the quantification of the expression of CD43, CD21, CD23 and CD11b. Data are individual values displayed as dots and the mean

±SEM (n = 10). Significances were determined with one-way ANOVA test with Tukey correction (black): $^{**}P < 0.01$; $^{***}P < 0.001$; $^{****}P < 0.0001$

Supplementary Figure 3: The effect of antibiotic treatment on the neonatal B lymphocytes. **(A)** Scheme of the antibiotic (Ab mix) treatment of pregnant females and pups after delivery (from E11.5 to D3 or D7), the collection of feces from the cages, and the extraction of spleen and lung samples. **(B)** Left figure, quantification of the sequenced V4 16s rDNA from the fecal samples in the cages from females and the litters exposed to antibiotics or not. Right figure, Shannon alpha diversity determination from the sequences (as dots and means, n = 5). **(C)** Left, Taxonomic bacterial composition (at a phylum level) of the fecal samples obtained from pregnant female mice treated with antibiotics or not (from E11 to D7 postnatal) and prepared and sequenced as indicated in the Materials and Methods. The data is presented as the relative frequency (n = 5 each group). Right, Frequency of Bacteroidota, Bacillota and Pseudomonadota in antibiotic-treated or untreated samples. The data are presented as box and whiskers plots of the median and range (n = 5 samples from different cages). **(D)** Violin graphs representing the biochemical analysis of glucose (left), LDH (middle) and albumin (right) from tissue homogenate supernatants from lung (top) and intestine (bottom); n = 11 (lung) and n = 4-5 (intestine). (**E**) Left, Scheme representing the antibiotics administering to the neonates is shown. The animals were sacrificed at D7 after delivery. Right, effect of antibiotic treatment on the frequency of $B220^{lo}$ cells relative to the B cells, the absolute number of $B220^{lo}$ and $B220^{hi}$ cells in D7 lung and spleen samples. **(F)** Left, the schematic representation of the experiment administering antibiotics to the pregnant females is shown. The pups were sacrificed at D1 after delivery. Some females were sacrificed at E18, and the embryos were dissected out, to obtain cell suspensions from lung samples. Right, effect of antibiotic treatment on the frequency of $B220^{lo}$ cells relative to the B cells (E18 and D1 samples), and the absolute number of $B220^{lo}$ and $B220^{hi}$ cells in D1 lung and spleen samples. The plots represent individual samples, and the lines are the mean ±SEM for two independent experiments (n = 6 for lung samples, and 2 for spleen samples). The data were compared with an unpaired *t*-test: $^{*}P < 0.05$; $^{**}P < 0.01$; $^{***}P > 0.001$; $^{****}P < 0.0001$.

Supplementary Figure 4: B220^{lo} and B220^{hi} cells from adult MyD88, TLR4 and TLR2/4 deficient mice. The number of B220^{lo} cells from adult mice in the lung (left, circles) and spleen (right, triangles) samples; BL6 mice in black, MyD88$^{-/-}$ mice in green, TLR2$^{-/-}$/TLR4$^{-/-}$ mice in blue, BL10 mice in grey and TLR4$^{-/-}$ mice in violet. **(A)** The results are expressed as relative to the B cells (% of B220^{lo}/B cells); **(B)** as the absolute cell number/organ of B220^{lo} cells and **(C)** of B220^{hi} cells. The data are displayed as dots, each one representing an individual value (n = 5-14), plotting the mean as a horizontal line and the SEM as vertical lines, and comparing the data with an unpaired Student's *t*-test: *$P < 0.05$; **$P < 0.01$; *** P>0.001.

Supplementary Figure 5: IL6 and IL10 production by CD138^{+} cells in D7 lung and spleen cells cultures activated with TLR2 and TLR4 ligands. IL6^{+} and IL10^{+} CD138^{+} cells (plasmablasts) were detected in the lung and spleen cultures as indicated in Material and Methods. **A) Representative plots of** IL6^{+} cells and IL10^{+} cells in spleen cultures. **B)** Comparison of the relative number of CD138^{+}IL6^{+} and CD138^{+}IL10^{+} cells present in the lung and spleen cultures. Right: Ratio of IL6^{+}/IL10^{+} CD138^{+} cells. Data are individual values displayed as dots and the mean ±SEM (n = 11-15). Significances were determined with one-way ANOVA test with Tukey correction (black) or unpaired t-Student (red): *$P < 0.05$; **$P < 0.01$; ****$P < 0.0001$.

Supplementary Figure 6. Infection i.n. of neonatal and adult mice with *Streptococcus pneumoniae* (SPN). **A)** Dose-response survival analysis in D14 C57BL/6 mice following infection with SPN (1 x 10^{5}–1 x 10^{6} CFU). Survival is presented as the percentage of surviving mice. **B)** Adult C57BL/6 and C57BL/10 mice were infected with 2 x 10^{6} CFU of SPN. At day 3 post-infection, lung and spleen samples were processed as described in the Materials and Methods to quantify the absolute number of CD19^{+} cells (n = 3 per dose for neonatal mice, and n = 5 for adult mice). Statistical significance was determined using one-way ANOVA with Tukey's post-hoc test: *$P < 0.05$**;** ****$P < 0.0001$**.**

Supplementary Table 1: List of Monoclonal Antibodies used

ANTIGEN	CLONE	ORIGIN/ ISOTYPE	FLUOROCHROME	COMPANY	TECHNIQUE
**CD3**	17A2	Rat IgG2b	FITC	Biolegend	FC
**CD5**	53-7.3	Rat IgG2a	Alexa-Fluor 647	Biolegend	FC
**CD9**	MZ3	Rat IgG2a	APC	Biolegend	FC
**CD11b (Mac-1)**	30-F11	Rat IgG2b	APC-Cy7	Biolegend	FC
**CD19**	6D5<br>1D3	Rat IgG2a<br>Rat IgG2a	BV421<br>PE	Biolegend<br>Invitrogen	FC
**CD21**	7EA	Rat IgG2a	BV510	Biolegend	FC
**CD23**	B3B4	Rat IgG2a	PE-Cy7	Biolegend	FC
**CD43**	S7<br>S11	Rat IgG2a<br>Rat IgG2b	BV711<br>PE	BD<br>Biolegend	FC
**CD44**	IM7	Rat IgG2b	BV510	Biolegend	FC
**CD45R (B220)**	RA3-6B2	Rat IgG2a	PeCy7	Biolegend	FC
**CD93**	AA4.1	Rat IgG2b	APC	Biolegend	FC
**CD138**	281-2	Rat IgG2a	APC	Biolegend	FC
**IgD**	11-26c.2a	Rat IgG2a	BV605	Biolegend	FC
**IgM**	RMM-1	Rat IgG2a	FITC	Biolegend	FC
**MHC-II (I-A/I-E)**	M5/114.15.2	Rat IgG2b	FITC	Invitrogen	FC
**IL6**	MP5-20F3	Rat IgG1	PE	Biolegend	FC
**IL10**	JES5-16E3	Rat IgG2b	PE	Invitrogen	FC
**CD16/CD32 "Fc Block"**	2.4G2	Rat IgG2b	Purificated	BD Biosciences	FC
**Isotype Controls**	RTK2758<br>MOPC-21	Rat IgG2a<br>Mouse IgG1	APC<br>Alexa Fluor 647<br>Biotin	Biolegend	FC

	RTK4530<br>RTK2071	Rat IgG2b<br>Rat IgG1	PE		
**Live/Dead Fixable**			BV510	ThermoFisher	

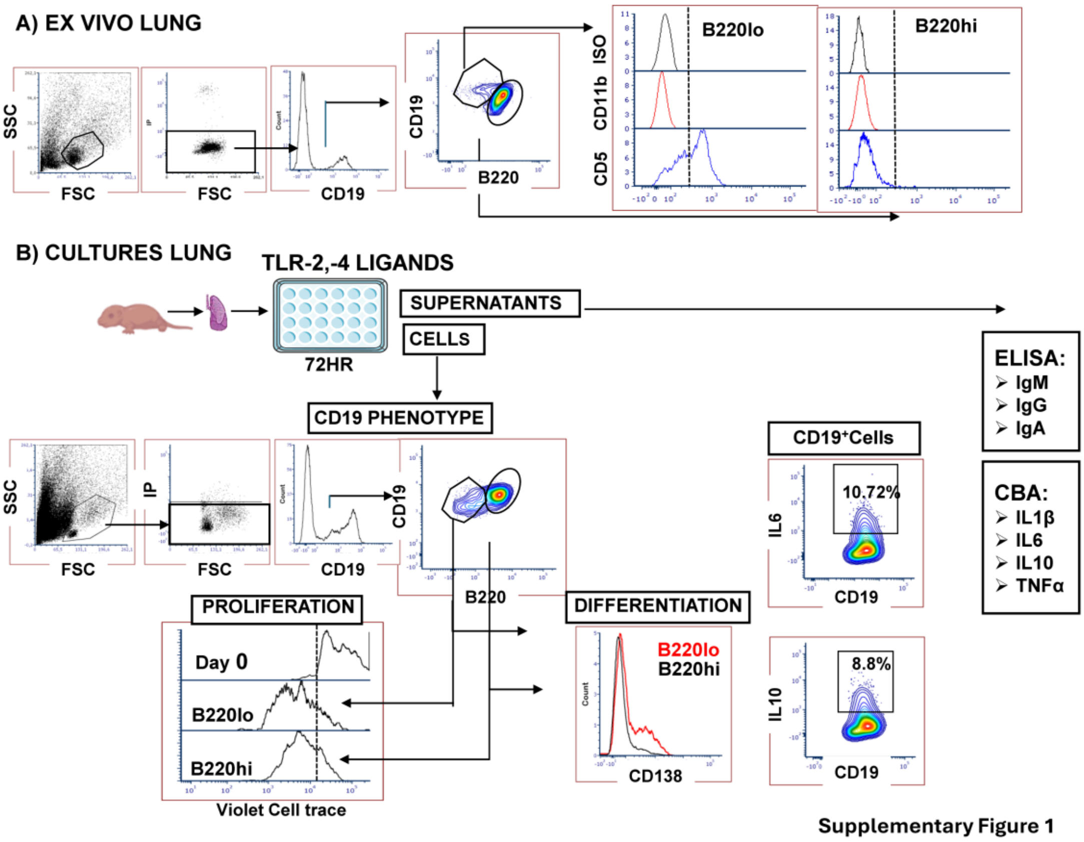

Supplementary Figure 1

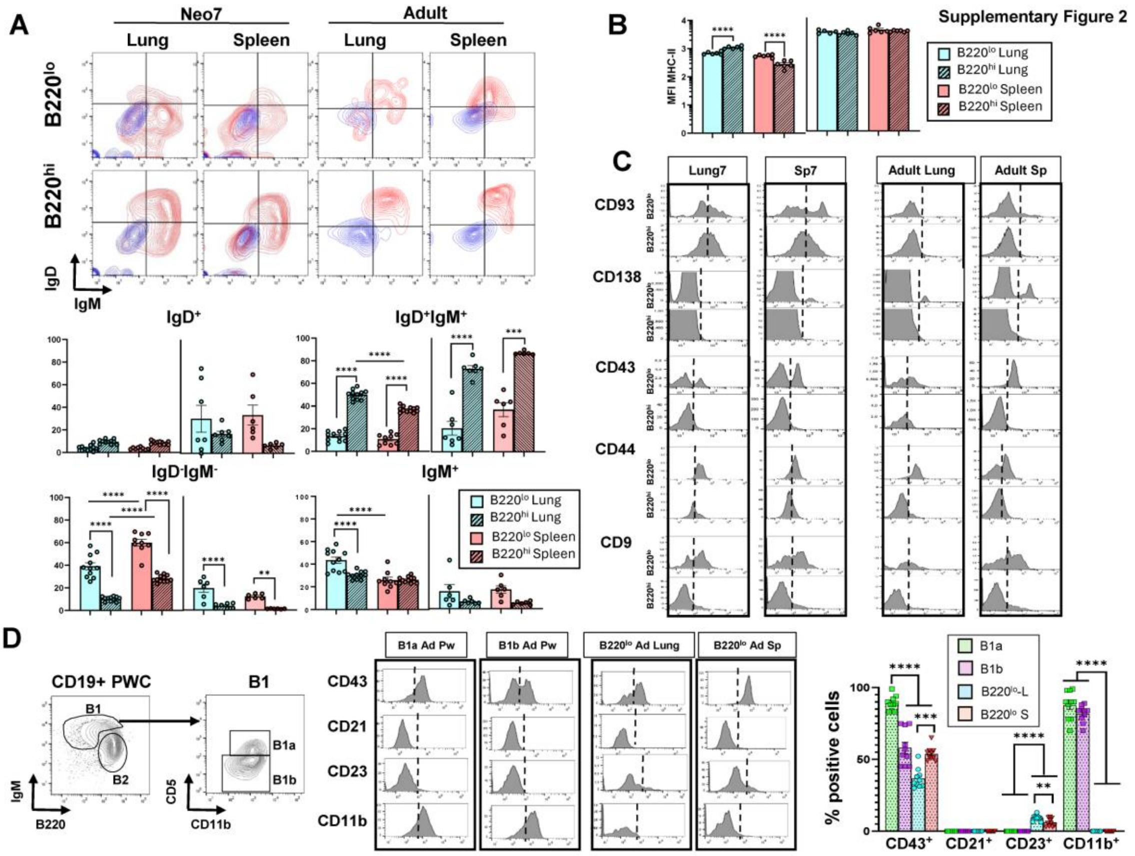
Supplementary Figure 2
A
Neo7
Adult
Lung
Spleen
B220lo
B220hi
IgD
IgM
IgD+
IgD+IgM+
IgD-IgM-
IgM+
B220lo Lung
B220hi Lung
B220lo Spleen
B220hi Spleen
B
MFI MHC-II
C
Lung7
Sp7
Adult Lung
Adult Sp
CD93
CD138
CD43
CD44
CD9
D
CD19+ PWC
B1
B2
B1a
B1b
CD5
CD11b
B220
B1a Ad Pw
B1b Ad Pw
B220lo Ad Lung
B220lo Ad Sp
CD43
CD21
CD23
CD11b
% positive cells
B220lo-L
B220lo S
CD43+
CD21+
CD23+
CD11b+

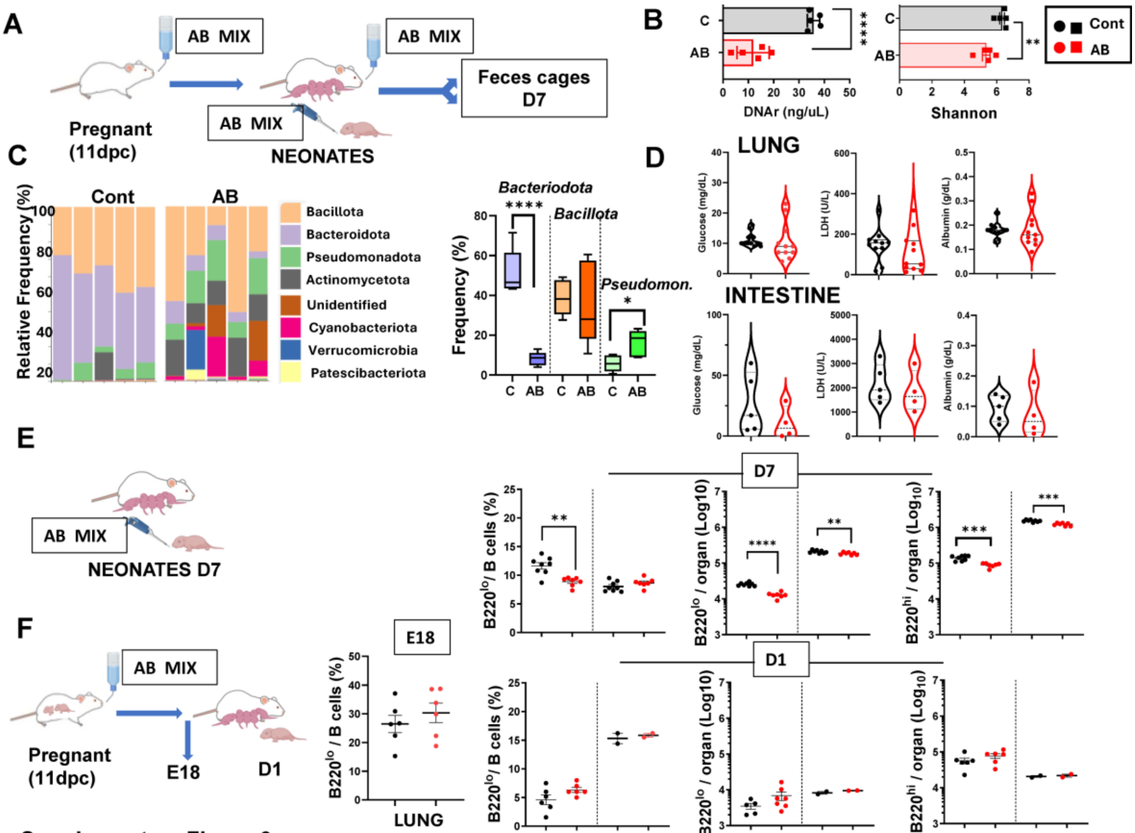

Supplementary Figure 3

Adult

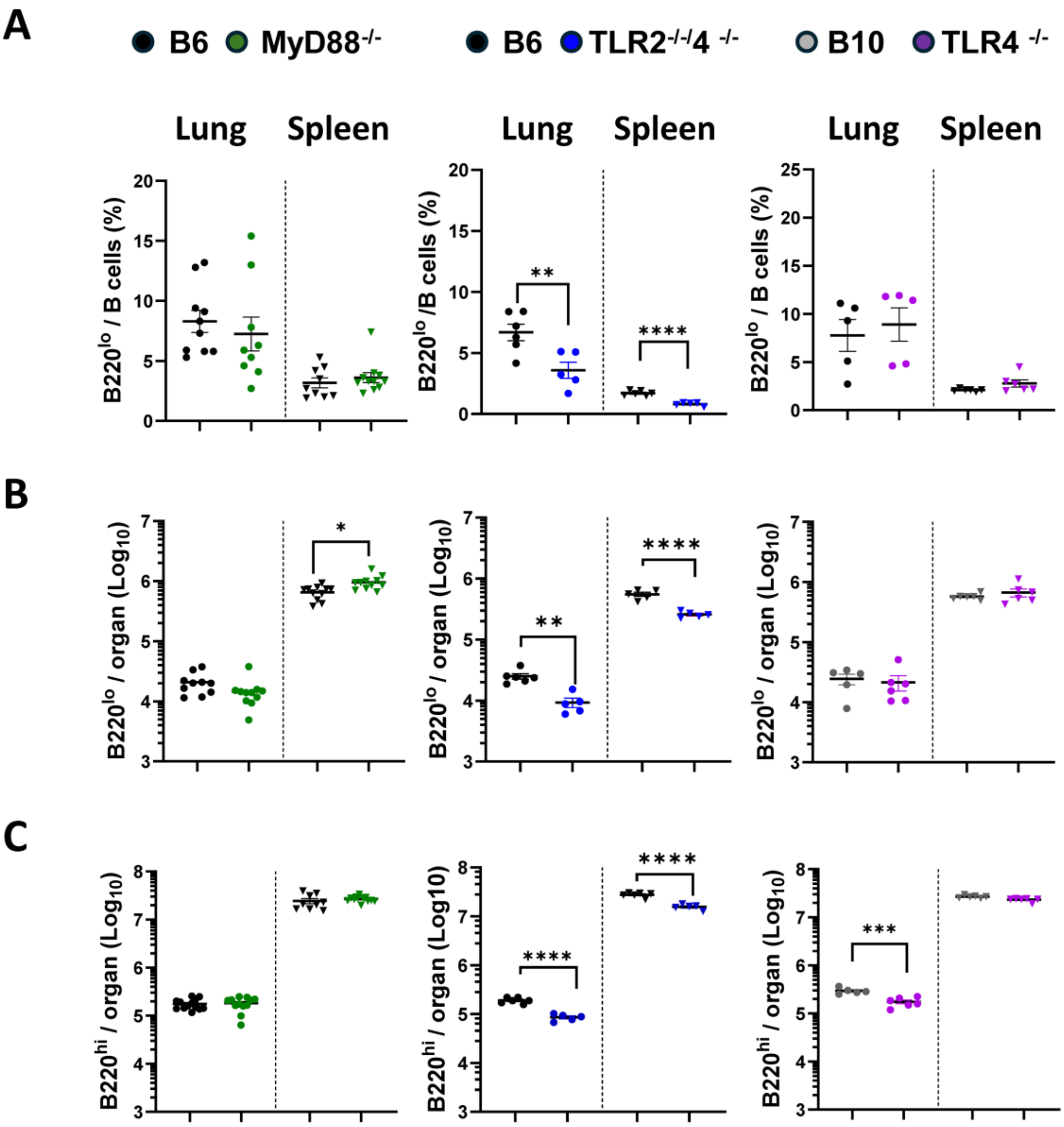

Supplementary Figure 4

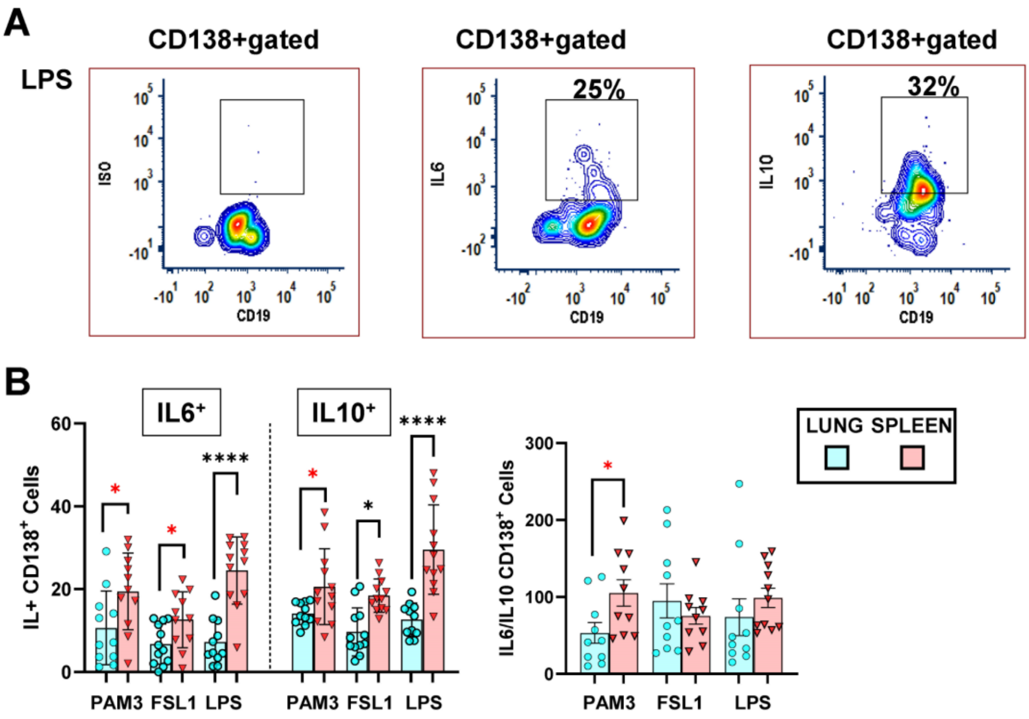

Supplementary Figure 5

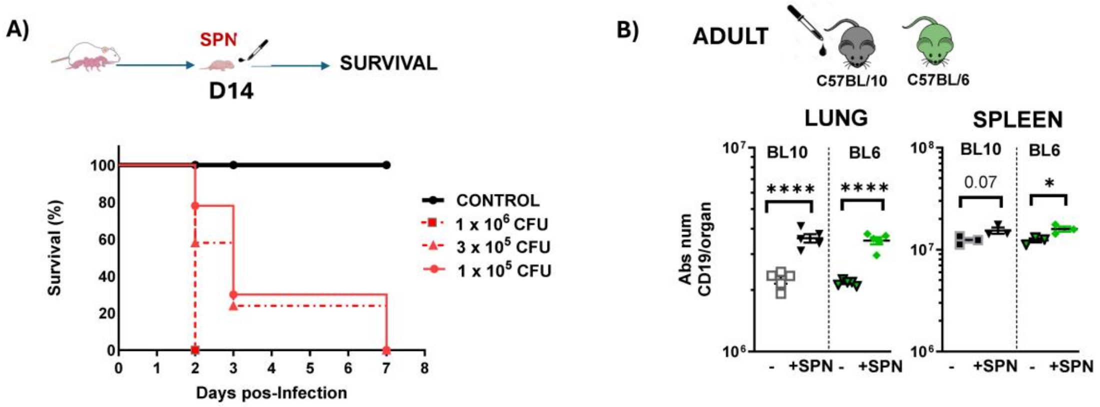

Supplementary Figure 6
